# EV-D68 virus-like particle vaccines elicit cross-clade neutralizing antibodies that inhibit infection and block dissemination

**DOI:** 10.1126/sciadv.adg6076

**Published:** 2023-05-17

**Authors:** Peter W. Krug, Lingshu Wang, Wei Shi, Wing-Pui Kong, Daniel L. Moss, Eun Sung Yang, Brian E. Fisher, Kaitlyn M. Morabito, John R. Mascola, Masaru Kanekiyo, Barney S. Graham, Tracy J. Ruckwardt

**Affiliations:** Vaccine Research Center, National Institute of Allergy and Infectious Diseases, NIH, Bethesda, MD 20892, USA.

## Abstract

Enterovirus D68 (EV-D68) causes severe respiratory illness in children and can result in a debilitating paralytic disease known as acute flaccid myelitis. No treatment or vaccine for EV-D68 infection is available. Here, we demonstrate that virus-like particle (VLP) vaccines elicit a protective neutralizing antibody against homologous and heterologous EV-D68 subclades. VLP based on a B1 subclade 2014 outbreak strain elicited comparable B1 EV-D68 neutralizing activity as an inactivated viral particle vaccine in mice. Both immunogens elicited weaker cross-neutralization against heterologous viruses. A B3 VLP vaccine elicited more robust neutralization of B3 subclade viruses with improved cross-neutralization. A balanced CD4^+^ T helper response was achieved using a carbomer-based adjuvant, Adjuplex. Nonhuman primates immunized with this B3 VLP Adjuplex formulation generated robust neutralizing antibodies against homologous and heterologous subclade viruses. Our results suggest that both vaccine strain and adjuvant selection are critical elements for improving the breadth of protective immunity against EV-D68.

## Teaser

An EV-D68 VLP vaccine based on a 2018 isolate provides robust cross-protection against viruses from heterologous subclades.

## INTRODUCTION

Enterovirus D68 (EV-D68), a non-polio respiratory enterovirus, was first identified as a cause of respiratory disease in the early 1960s (*1*). Since then, EV-D68 has caused clusters of severe respiratory disease in young children during the late summer of 2014, 2016, and 2018 (*2*–*6*). In a small fraction of infected children, acute flaccid myelitis (AFM), paralysis due to spinal cord gray matter injury, presents after resolution of the respiratory infection (*7*). Cementing the causal link between EV-D68 and paralytic disease, autopsy samples from a child that died of AFM were used to demonstrate EV-D68 genomic RNA and protein in anterior horn motor neurons in the cervical spinal cord (*8*). Furthermore, this evidence suggests that inhibiting viral dissemination from the lung could prevent paralytic disease manifestation. Coronavirus disease 2019 mitigation efforts stifled the spread of EV-D68 and other respiratory viruses in 2020 (*9*), but increases in EV-D68 detection in many parts of the world in 2021 (*10*, *11*) indicate that future outbreaks are likely. Cases of severe respiratory infection have increased in the late summer of 2022 (*12*).

Treatments for patients acutely affected by EV-D68 during the severe respiratory disease phase remain supportive, and for those that progress to AFM, treatments such as steroids and human gamma globulin have been used with limited success (*13*, *14*). In animal models of EV-D68 disease, monoclonal antibodies and human gamma globulin can protect against paralysis if given either before or soon after infection (*15*–*17*). These studies suggest a narrow window for diagnosis and treatment to mitigate AFM in humans, and government authorities have urged vigilance to recognize early symptoms of the disease (*18*). Limited data on clinical treatment of EV-D68 infection and the potential for severe and long-lasting sequelae warrant the development of vaccines to prevent both severe respiratory disease and AFM.

Approaches for vaccination against EV-D68 can leverage the considerable research and development for picornavirus vaccines over the last century. Inactivated poliovirus vaccine (IPV) is highly effective at preventing disseminated diseases, and the live-attenuated poliovirus vaccine (OPV) can also provide mucosal immunity to prevent replication of wild poliovirus (PV) in the gastrointestinal tract (*19*). These two vaccines have been instrumental in the near-complete eradication of wild-type PV (*20*), and recent advancements in OPV safety should protect against reversion to virulence leading to vaccine-derived disease (*21*). Inactivated vaccines have also had considerable success in preventing the spread of foot-and-mouth disease virus (FMDV); however, vaccination against one serotype does not provide protection across the other six viral serotypes. Multivalent inactivated vaccines are used to extend protection to circulating FMDV strains because success in creating cross-serotype vaccines has been limited (*22*). For all known picornavirus vaccines, neutralizing antibodies are the correlate of protection, emphasizing their priority in vaccination development for EV-D68 (*23*). These antibodies bind epitopes in exposed loops on the picornavirus capsid that are hotspots for mutation, and this evolution drives virus escape from host humoral immunity (*24*, *25*). EV-D68 variability at these epitopes has led to the divergence of virus clades based on capsid protein sequences (*26*), and following the nomenclature of viral phylogeny [as described visually on the Nextstrain EV-D68 internet site (fig. S1) (*27*)], the known circulating strains in 2022 are exclusively B3 and A2 subclade viruses.

Virus-like particle (VLP) vaccines have been developed for many human and animal picornaviruses (*28*–*33*). VLP platforms have the advantage of using viral sequences from clinical samples to express capsid and protease genes, and VLP vaccines can be rapidly produced. VLPs avoid cell culture adaptation that can occur during preparation of inactivated virus (*34*) and eliminate the risk that incomplete inactivation could cause outbreaks of disease (*35*, *36*). Here, we demonstrate that antibodies elicited by vaccination with an EV-D68 VLP vaccine have strong neutralizing activity in vitro, and passive antibody transfer prior to intranasal infection can abrogate mouse-adapted EV-D68 replication and dissemination in vivo. Furthermore, we evaluated the immunoglobulin G (IgG) subclass profile elicited by B3 VLP formulated with a variety of adjuvants and determined the cross-clade and cross-subclade neutralization capacity of the VLP-elicited antibody from mice and nonhuman primates (NHPs). Our results, using different virus strains and adjuvants, have implications for the design and implementation of VLP vaccines for EV-D68.

## RESULTS

### B1 subclade immunogens elicit a strong heterologous neutralizing antibody

**Fig. 1.**
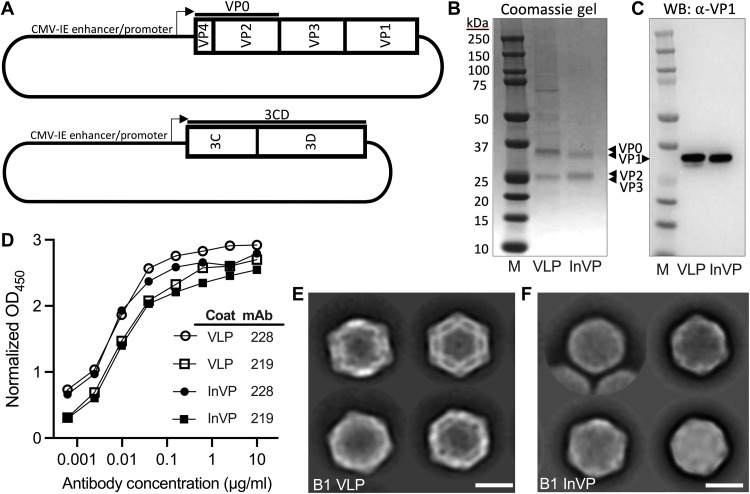
B1 EV-D68 VLPs are antigenically similar to inactivated B1 EV-D68 viral particles. (**A**) Plasmid constructs expressing P1 and 3CD polypeptides based on the sequences from the EV-D68 US/CO/2014-93 isolate for VLP production. (**B**) Protein gel of EV-D68–purified VLP (VP0, VP1, and VP3) and β-propiolactone–inactivated EV-D68 US/MO/2014-18947 viral particles (InVP; VP1, VP2, and VP3). M, protein marker. (**C**) Western blot (WB) probed with a commercial anti–EV-D68 VP1 (α-VP1) monoclonal antibody. (**D**) EV-D68 monoclonal antibody detection of VLP and InVP antigens by direct ELISA. Open symbols, plates coated with 100 ng of VLP; closed symbols, plates coated with 100 ng of InVP. OD_450_, optical density at 450 nm. Human monoclonal antibodies (mAb) 228 and 219 were a gift from J. Crowe at Vanderbilt University and are described in (*16*). (**E** and **F**) Negative-stain electron microscopy of EV-D68 VLP (E) and InVP (F). Scale bars, 20 nm.

Picornavirus vaccines have traditionally used inactivated virus to elicit neutralizing antibody to prevent disseminated disease, as demonstrated for PV and FMDV vaccines. We purified B1-based β-propiolactone–inactivated virus particles (InVP) from lysates of US/MO/2014-18947–infected rhabdomyosarcoma (RD) cell culture as described in Materials and Methods and table S1. B1-based VLPs were made by transfecting mammalian cells with plasmids expressing the P1 capsid and 3CD protease of EV-D68 US/CO/2014-93 (Fig. 1A) and purified as described in Materials and Methods. SDS–polyacrylamide gel electrophoresis (SDS-PAGE) analysis demonstrated protein bands corresponding to VP0, VP1, and VP3 between 25 and 37 kDa in VLP, while VP1, VP2, and VP3 were present in InVP (Fig. 1B). VP1 was detected in both purified VLP and InVP using a commercially available α–EV-D68 VP1 antibody in Western blot (Fig. 1C) and similarly bound previously published EV-D68 antibodies mAb228 and mAb219 (*16*) by enzyme-linked immunosorbent assay (ELISA) (Fig. 1D). Negative-stain electron microscopy of both B1 VLP (Fig. 1E) and B1 InVP (Fig. 1F) demonstrated particles of hexagonal appearance approximately 30 nm in diameter as expected for picornavirus mature and empty particles (*37*).

**Fig. 2.**
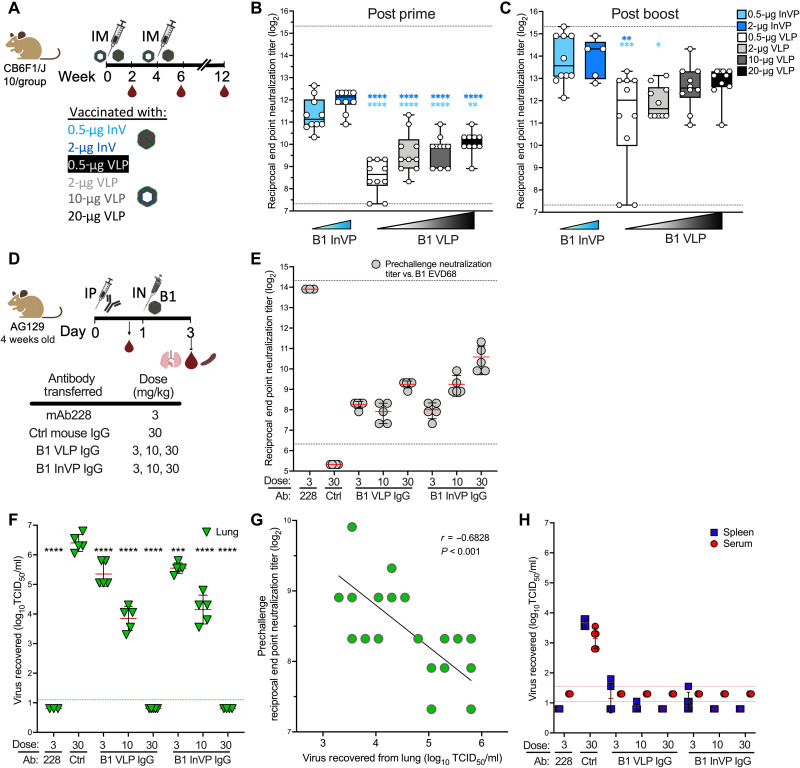
B1 VLP and B1 InVP elicit potent neutralizing antibody that blocks EV-D68 in cell culture and abrogates lung replication and dissemination in vivo. (**A**) Vaccination schema. Serum was obtained on weeks 2, 6, and 12. IM, intramuscularly. (**B** and **C**) Neutralization of EV-D68 US/MO/2014-18947 at week 2 (B) or week 6 (C). Dotted lines indicate limits of detection; error bars indicate ±SD. Ordinary one-way analysis of variance (ANOVA) with Tukey’s multiple comparisons test was used to compare neutralizing titers at each time point. Light blue and dark blue asterisks indicate comparison of the VLP to 0.5 μg of InVP and 2.0 μg of InVP, respectively. (**D**) Antibody transfer study schema. IgG from VLP- or InVP-vaccinated mice at week 12 were purified and injected intraperitoneally (IP) into AG129 mice at the indicated concentration (*n* = 3 to 5 mice per group). Serum was taken to confirm the transfer of neutralizing antibodies. IN, intranasally. (**E**) B1 subclade EV-D68 US/MO/2014-18947 end point neutralization titers before virus challenge. Dotted lines indicate limits of detection; error bars indicate ±SD. Mice were challenged intranasally with 10^4.4^ median tissue culture infectious dose (TCID_50_) B1 subclade EV-D68 Mp40. Two days after challenge, lungs, spleen, and serum were taken for assessment of viral load. (**F**) Viral lung titers demonstrate an antibody dose-dependent decrease in virus replication in the lung. Ordinary one-way ANOVA with Dunnett’s multiple comparisons test was used to determine significance relative to the naïve IgG control (30 mg/kg). The dotted line indicates the lower limit of detection in the assay; error bars indicate ±SD. (**G**) Correlation of lung viral load and serum end point neutralization titer in pre-challenge serum samples. (**H**) Viral titers, measured by TCID_50_ assay, demonstrate that IgG from vaccinated mice inhibit mouse-adapted EV-D68 dissemination to the spleen and serum. Blue and red dotted lines indicate the limit of virus detection in spleen and serum, respectively; error bars indicate ±SD. *P < 0.05, **P < 0.01, ***P < 0.001, and ****P < 0.0001.

To compare the ability of VLP and InVP to elicit neutralizing antibodies, CB6F1 mice were immunized twice in a 4-week interval with various concentrations of each immunogen formulated with Sigma Adjuvant System (SAS; see schema in Fig. 2A). Two weeks after prime, neutralizing antibody responses were measured using EV-D68 US/MO/2014-18947, the same strain as the B1 InVP (see table S1 for differences). Neutralizing activity elicited by B1 InVP was significantly higher than that elicited by an equivalent dose of B1 VLP (*P* < 0.001; Fig. 2B). By 2 weeks after boost, animals immunized with 10 and 20 μg of VLP had comparable serum neutralizing activity to animals vaccinated with InVP (Fig. 2C), indicating that both VLP and InVP can elicit similar levels of neutralizing responses after two immunizations in mice.

### B1 subclade–elicited IgG blocks homologous EV-D68 lung replication and dissemination in mice

To assess the ability of vaccine-elicited antibodies to protect against EV-D68 infection, 3- to 30-μg doses of IgG purified from VLP- or InVP-immunized CB6F1 mice were passively transferred into interferon-αβγ (IFN-αβγ) receptor knockout (AG129) mice (Fig. 2D). To evaluate protection from respiratory replication and dissemination, we infected mice intranasally with mouse-adapted B1 subclade virus (table S2) (*38*). This virus, B1 18949 Mp40, reaches peak titers in the lung at day 2 postinfection and disseminates to the blood and spleen (fig. S2A). Control mouse IgG and mAb228 were used as negative and positive controls, respectively, and sera were obtained 20 hours after transfer to measure neutralizing activity before challenge (Fig. 2E). Mice were euthanized 2 days after challenge, and virus in the lung, spleen, and serum was measured by tissue culture infectious dose (TCID_50_) assay. Lung replication was reduced in a dose-dependent manner, with less virus detected in the lungs of mice receiving 3 and 10 mg/kg of VLP- or InVP-immune IgG and no detectable virus at a higher dose of 30 mg/kg of immune IgG, resulting in a 5-log reduction compared to the mice that received control IgG (*P* < 0.0001; Fig. 2F). Pre-challenge neutralization titers inversely correlated with lung viral load (Pearson *r* = −0.68; Fig. 2G), and there was no significant difference in the reduction of viral load at any specific IgG dose comparing InVP-elicited IgG to VLP-elicited IgG. Dissemination into the blood was reduced below the limit of detection in all animals that received any dose of VLP- or InVP-elicited IgG, and virus detection in the spleen was markedly reduced or undetectable (Fig. 2H). These data demonstrate that B1 VLP– and InVP-elicited antibodies protect against virus infection and dissemination after intranasal challenge with homologous EV-D68 in mice.

### Antibodies elicited by B1 subclade immunogens exhibit limited cross-clade neutralization

**Fig. 3.**
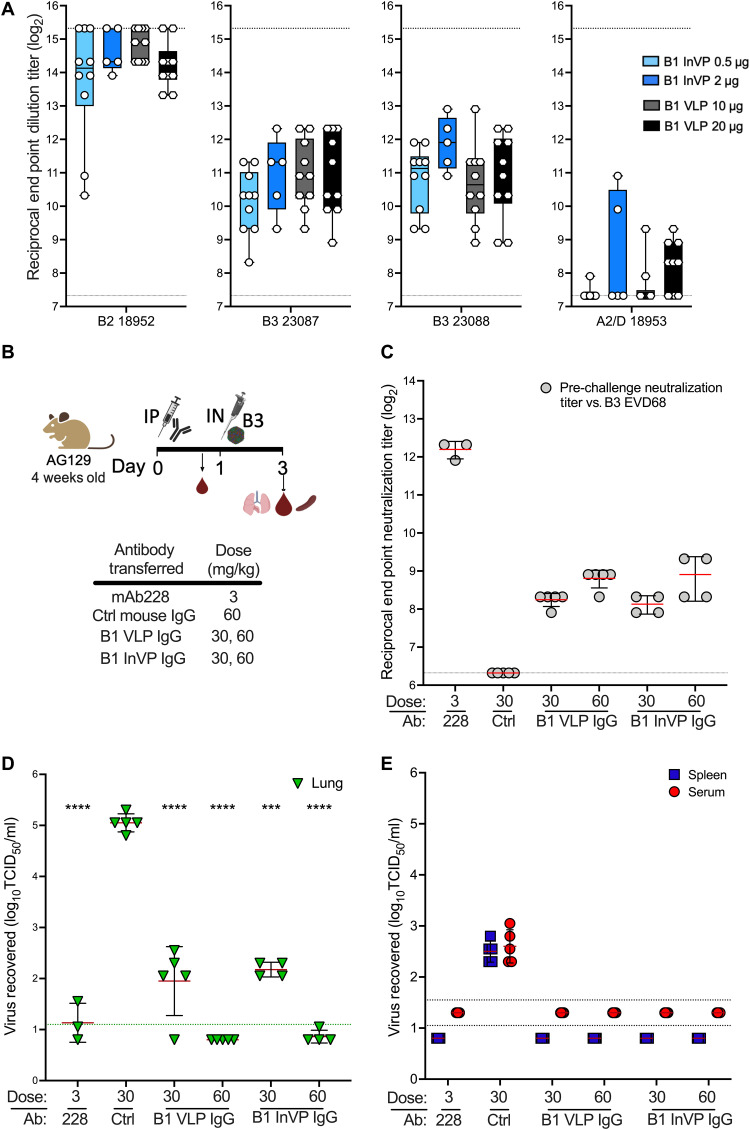
B1 VLP and InVP vaccination elicited antibodies with reduced capacity to neutralize heterologous EV-D68 strains in cell culture and require a higher transfer dose to abrogate virus replication in vivo. (**A**) Cross-subclade neutralization of EV-D68 with serum antibodies elicited by B1 subclade immunogens. Week 6 serum samples from mice vaccinated with the indicated B1 immunogen (described in Fig. 2A) were used to neutralize the indicated EV-D68 viruses. *n* = 5 to 10 mice per group. Each serum sample was tested in duplicate in two assays. Dotted lines indicate the limits of detection in the assay; error bars indicate ±SD. (**B**) Passive transfer schema. AG129 mice were administered the indicated antibody at the indicated dose via intraperitoneal injection. Eighteen hours later, serum was taken to confirm the transfer of neutralizing antibodies against B3 subclade EV-D68. Four hours later, the mice were challenged intranasally with 10^4.2^ TCID_50_ B3 subclade mouse adapted EV-D68 Mp9. Two days postinfection, lungs, spleen, and serum were taken for assessment of viral load. (**C**) B3 subclade EV-D68 US/2018-23087 pre-challenge endpoint neutralization titers. Dotted lines indicate upper and lower limits of detection; error bars indicate ±SD. (**D**) IgG dose-dependent inhibition of B3 EV-D68 replication in mouse lungs. The green dashed line indicates the limit of detection in lung; error bars indicate ±SD. (**E**) Abrogation of EV-D68 dissemination to spleen and blood by α–EV-D68 passive transfer antibodies. Blue and red dashed lines indicate the limit of detection in spleen and serum, respectively; error bars indicate ±SD. ***P < 0.001 and ****P < 0.0001.

At the initiation of our studies, we based the vaccine on B1 subclade virus because only EV-D68 isolates from 2014 and earlier were available; however, EV-D68 has undergone considerable genetic drift since 2014 (fig. S1). B1 subclade viruses have been largely replaced by viruses in the B3 subclade in 2016 and 2018 (*2*, *4*). Thus, we next considered cross-neutralization of currently circulating EV-D68 B3 and A2 subclades, as some monoclonal and polyclonal EV-D68 antibodies have been found to exhibit reduced cross-reactivity (*16*, *32*, *39*). Sera from mice vaccinated with B1 VLP and B1 InVP were assessed in vitro for neutralization of isolates from the B2, B3, and A2 subclades (Fig. 3A and table S1). B1 VLP– and B1 InVP–elicited antibodies comparably neutralized all viruses in the panel. B2 virus was neutralized well, but neutralization of viruses from the 2018 circulating B3 subclade were four- to sixfold reduced compared to homologous B1. More notably, neutralization of an A2 subclade virus was reduced 20- to 40-fold compared to B1, raising concern about the utility of B1 immunogens to elicit neutralizing antibody against heterologous subclades of EV-D68, particularly A2 subclade viruses.

### B1 vaccine–elicited IgG inhibits B3 mouse-adapted virus in vivo

Next, we passively transferred the purified IgG elicited by the B1 VLP or B1 InVP 1 day before challenge with a B3 subclade mouse-adapted virus to determine whether antibodies elicited by B1 immunogens could protect against B3 subclade EV-D68 in vivo (Fig. 3B). We first evaluated neutralization against B1 and B3 viruses using purified polyclonal IgG elicited by B1-based VLP or InVP and found a three- to fourfold reduction in the neutralization of B3 subclade virus compared to B1 (fig. S3). We therefore increased the dose of passively transferred antibodies up to 60 mg/kg and assessed end point neutralization titers in sera obtained 18 hours after transfer (Fig. 3C). To evaluate protection following intranasal infection, we adapted a B3 subclade virus for growth in AG129 mouse lungs (EV-D68 B3 23087 Mp9 or “B3 Mp9”; table S2) as described in Materials and Methods. B3 Mp9 replicates to peak titers in mice 2 days after intranasal infection (fig. S2B). To evaluate antibody-mediated protection against replication and dissemination, the recipients of passively transferred antibody were challenged intranasally with B3 Mp9, and lung, spleen, and serum were harvested for virus isolation 2 days after infection. In contrast to the B1 challenge, a dose of 30 mg/kg of purified IgG reduced viral load by 3 log_10_ in the lung (*P* ≤ 0.001) but was insufficient to prevent viral replication of B3 Mp9 in the lung, with full protection achieved only at the dose of 60 mg/kg (Fig. 3D). However, virus was not detected in spleen and serum at antibody doses of either 30 or 60 mg/kg, indicating protection of virus dissemination from the lung to other organs (Fig. 3E). Antibodies elicited by either B1 VLP or B1 InVP had no significant difference in lung titer reduction at any dose. Together, these data indicate that the B1 VLP and B1 InVP vaccines can elicit cross-protective responses against B3 infection that inhibit dissemination, even if they do not fully abrogate viral replication in the lungs.

### A B3 subclade VLP vaccine elicits potent neutralizing antibody that blocks homologous virus in vivo

**Fig. 4.**
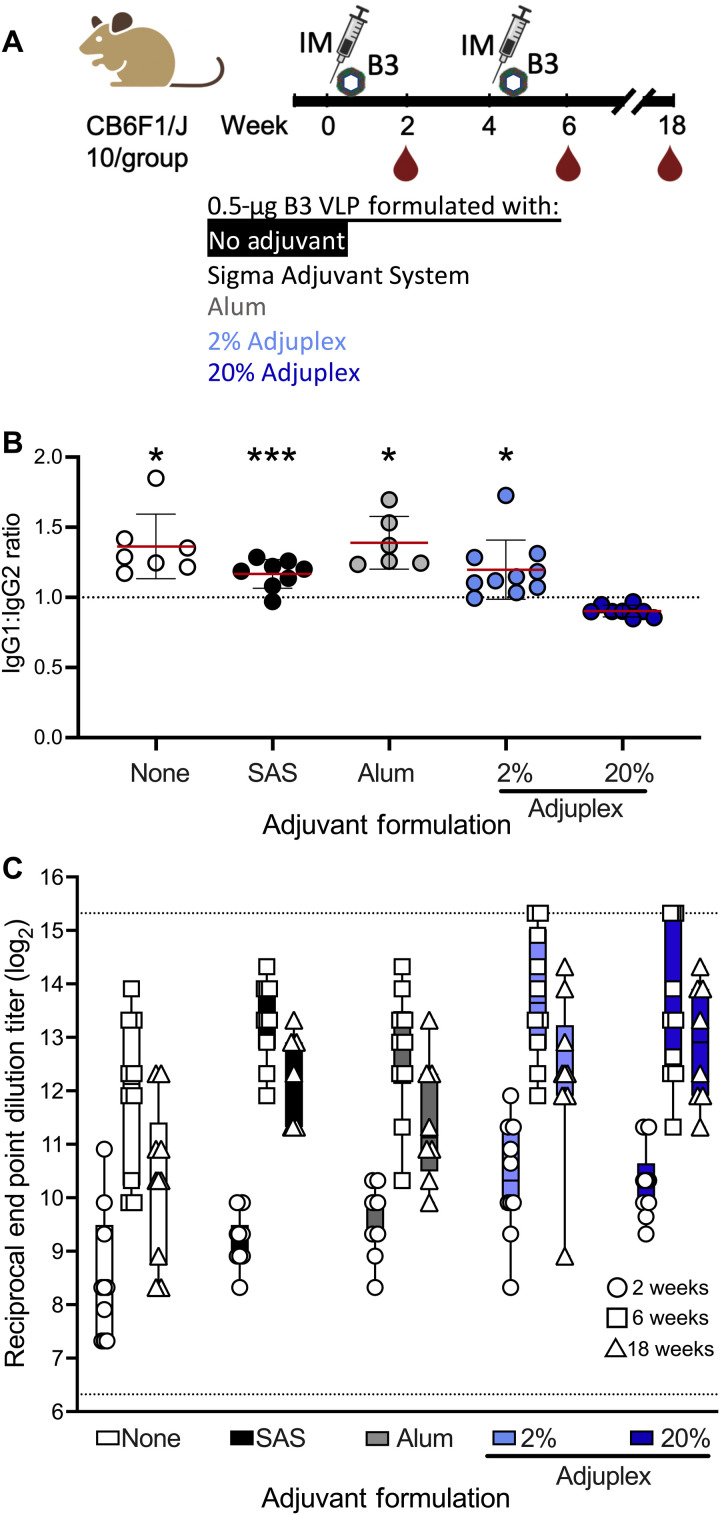
B3 EV-D68 VLP vaccine formulations elicit robust and durable neutralizing antibody. (**A**) B3 VLP vaccination schema. Ten mice per group were vaccinated and boosted intramuscularly with the indicated formulation of adjuvant and B3 VLP. Sera were sampled 2, 6, and 18 weeks after prime vaccination (−2, 2, and 14 weeks after boost vaccination). (**B**) 20% Adjuplex-formulated B3 VLP stimulates a balanced T_H_ response, indicated by the ratio of IgG1 to IgG2a determined by an IgG subclass–specific ELISA. Fourteen days after boost sera were used to bind to EV-D68 VLP coated on ELISA plates and reacted with anti-IgG1 or anti-IgG2a secondary antibodies as described in Materials and Methods. Samples with undetectable IgG2a were omitted from the comparison; see fig. S4 for details. (**C**) Magnitude and durability of neutralizing antibody elicited from B3 VLP vaccine formulations. The indicated sera were used to neutralize EV-D68 US/2018-23087. Serum samples were tested in duplicate in two individual assays. At each time point, ANOVA with Tukey’s multiple comparisons test was used to compare the significance between the neutralizing titers elicited from each B3 VLP formulation. Horizontal dotted lines indicate the upper and lower limits of detection in the assay; error bars indicate ±SD. *P < 0.05 and ***P < 0.001.

Because B1 VLP elicited lower neutralizing responses against B3 and A2 subclade EV-D68 viruses and afforded less protection against B3 subclade viral challenge, the flexibility of the VLP production platform was leveraged to prepare B3 VLP that was based on a 2018 outbreak strain US/MD/2018-23209 (fig. S4). A low dose (0.5 μg) of the B3 VLP was tested alone or in formulation with different adjuvants in a prime/boost vaccination regimen with a 4-week interval (Fig. 4A). We first evaluated the IgG subclass, measuring the IgG2a/c and IgG1 B3 VLP–binding antibody as surrogates of T helper 1 (T_H_1) and T_H_2 immunity, respectively, and calculated an IgG1/IgG2a ratio to control varying levels of EV-D68–specific antibody (Fig. 4B and fig. S5). Formulation with 20% Adjuplex elicited a more balanced response, indicated by a lower EV-D68–specific IgG1/IgG2a ratio than other groups (*P* < 0.02; Fig. 4B). Notably, decreasing the concentration of Adjuplex from 20 to 2% resulted in an increased ratio of IgG1/IgG2a. We also assessed the capacity of these sera to neutralize homologous B3 virus through week 18. Serum neutralizing activity was robust and durable across all adjuvanted B3 VLP formulations, and there were no significant differences between adjuvants at any time point (Fig. 4C). Overall, these data suggest that the carbomer-lecithin adjuvant Adjuplex elicits a more balanced T_H_1/T_H_2 response and as potently elicits neutralizing activity as the aluminum hydroxide–based Alhydrogel and the monophosphoral lipid A–containing oil-in-water research adjuvant SAS. These data indicate that while adjuvant choice did not affect the overall neutralizing activity, it did affect the T_H_1/T_H_2 balance of the immune response.

**Fig. 5.**
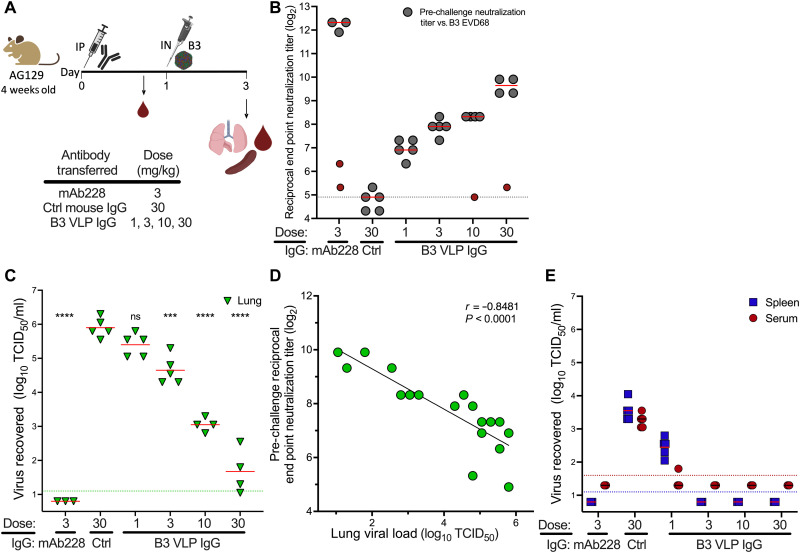
B3 VLP Adjuplex formulations elicit neutralizing antibody that blocks B3 virus replication and dissemination in vivo. (**A**) Passive transfer schema. Week 18 sera from mice vaccinated with 2 and 20% Adjuplex/B3 VLP formulations were pooled, and the IgG was purified. AG129 (*n* = 5) mice were administered IgG at the indicated dose via intraperitoneal injection. Serum was obtained to confirm antibody transfer and then the mice were challenged with 10^4.5^ TCID_50_ B3 EV-D68 Mp20. (**B**) Pre-challenge serum neutralization titers against EV-D68 B3 2018-23087. The dotted line indicates the limit of detection; error bars indicate ±SD. Red symbols represent mice that were omitted from the analysis because of very low neutralization titer in the pre-challenge sera. (**C**) Dose-dependent inhibition of EV-D68 replication in mouse lung. ANOVA with Dunnett’s multiple comparisons test was used to compare the virus recovered from lung in each group compared to the naïve IgG negative control group. The green dotted line indicates the limit of virus detection in the lung; error bars indicate ±SD. (**D**) Pearson correlation demonstrates that serum α–EV-D68 VLP antibody titer is indirectly proportional to virus replication in the lung. (**E**) α–EV-D68 VLP antibody transfer prevents dissemination of mouse-adapted virus to the spleen and blood. Red and blue dotted lines denote the lower limit of detection in serum and spleen, respectively. Error bars indicate ±SD. ns, *P* > 0.05; ***P < 0.001 and ****P < 0.0001.

Total IgG was purified from pooled week 18 sera from B3 VLP Adjuplex–adjuvanted mice and passively transferred into AG129 1 day before challenge with B3 EV-D68 (Fig. 5A). Again, naïve mouse IgG and mAb228 were used as controls, and sera were obtained before challenge to demonstrate successful antibody transfer (Fig. 5B). To better assess protection, a B3 virus, B3 23087 Mp20 (“B3 Mp20”; fig. S2C and table S2) was generated to achieve higher virus replication levels in the lungs, blood, and spleen by serially passaging the B3 Mp9 virus an additional 11 times through AG129 mouse lungs. Despite this increase in dissemination from the lung, the B3 Mp20 virus was restricted from entering the central nervous system (CNS; fig. S2D). Following B3 Mp20 intranasal challenge of B3 VLP IgG passively transferred mice, we observed an IgG dose-dependent inhibition of viral lung replication (Fig. 5C). Virus recovered from the lungs inversely correlated with post-transfer neutralizing antibody (Pearson *r* = −0.85; Fig. 5D). Dissemination into the blood and spleen was detectable only at the lowest dose of IgG transferred (1 mg/kg), indicating complete protection from dissemination at doses at or above 3 mg/kg (Fig. 5E). These data demonstrate that antibodies elicited by a B3 VLP vaccine are protective against homologous challenge.

### B3 VLP vaccination elicits strong cross-clade neutralizing antibody in mice and NHPs

**Fig. 6.**
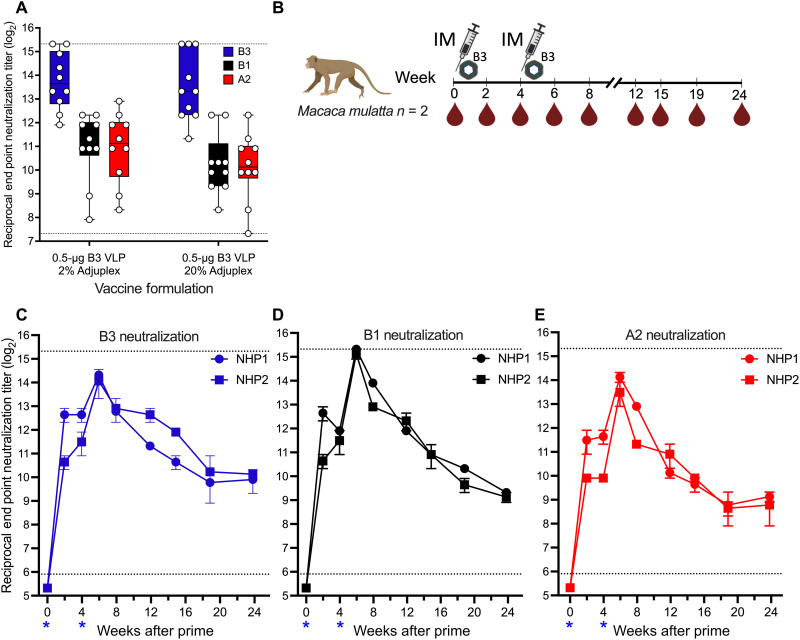
Cross-clade neutralizing antibody elicited from B3 VLP vaccination in mice and NHPs. (**A**) Cross-neutralization of EV-D68 viruses with mouse sera raised against B3 VLP formulated with 2 or 20% Adjuplex from the experiment described in Fig. 4A. Sera were used to neutralize the following viruses: B3 USA/2018-23087 (blue boxes), B1 US/MO/2014-18947 (black boxes), and A2 US/KY/2014-18953 (red boxes). Dashed lines indicate the upper and lower limits of detection; error bars indicate ±SD. (**B**) NHP vaccination schema. Two rhesus macaques were primed (week 0) and boosted (week 4) with 50 μg of B3 VLP formulated with 20% Adjuplex. Serum samples were acquired on the indicated weeks after prime vaccination and used to neutralize the homologous B3 subclade USA/2018-23087 virus (**C**) (blue), the B1 subclade heterologous US/MO/2014-18947 virus (**D**) (black), and the A2 subclade heterologous US/KY/2014-18953 virus (**E**) (red) as described in Materials and Methods. Each point represents the mean neutralization titer from two assays, and error bars reflect the range of the individual replicates. Blue asterisks on horizontal axes indicate the dates of vaccination.

To assess the capacity of the 20% Adjuplex-adjuvanted B3 VLP to elicit cross-clade neutralizing antibody, sera obtained 2 weeks after the boost were used to neutralize B3, B1, and A2 subclade viruses. B3 VLP vaccination elicited similarly high levels of neutralizing antibodies against the A2 and B1 subclades (1.1-fold difference; Fig. 6A), while those titers were about sevenfold lower compared to the B3 homologous virus. This profile was notably different from the B1 VLP cross-neutralization profile (10-fold difference between B3 and A2 subclades; Fig. 3A). This difference was not an adjuvant effect as SAS-formulated B3 VLP also elicited better A2 subclade–specific neutralizing antibodies compared to similarly adjuvanted B1 VLP (fig. S6).

To evaluate the magnitude, durability, and cross-clade neutralization elicited by B3 VLP in NHPs, two rhesus macaques were primed and boosted in a 4-week interval with 50 μg of B3 VLP formulated with 20% Adjuplex (Fig. 6B). Sera from these macaques showed high neutralizing activity against all three subclade viruses after a single immunization (Fig. 6, C to E). Serum neutralization titers were boosted 8- to 16-fold by the second B3 VLP immunization, and neutralizing activity against all three subclade viruses was maintained through 6 months after prime. Vaccinated NHP sera showed better cross-clade neutralization (2.1-fold difference for B1 and 1.2-fold difference for A2 compared to B3 at week 6) than that of mice. Together, our data demonstrate that a B3 subclade–based VLP is a promising vaccine candidate to help mitigate current and future outbreaks of EV-D68.

## DISCUSSION

EV-D68 is a serious threat to human health, causing severe respiratory disease in children (*40*) and in older adults (*5*, *41*). Increases in pediatric AFM cases have followed EV-D68 outbreaks since 2014. There are currently no approved therapeutic interventions and EV-D68 continues to cause seasonal outbreaks. Having a safe and effective vaccine that is ready is critical for preventing severe respiratory infection and managing longer-term neurological sequelae in the event of a wider epidemic. To this end, we demonstrate that VLP vaccination elicits potent neutralizing antibody that confers protection from systemic virus dissemination in an in vivo challenge model. Our results suggest that VLPs based on different EV-D68 subclades can elicit distinctive cross-neutralization capacity and that adjuvant formulation can have a substantial effect on the IgG subclass balance of the vaccine-induced immune response. Together, informed strain selection and VLP vaccine formulation decisions will drive safe and effective vaccine design for future EV-D68 outbreaks.

As EV-D68 infects humans via the respiratory tract, we used an intranasal infection model (*38*) with mouse-adapted viruses described here to model EV-D68 lung replication in humans and to determine the capability of vaccine-elicited neutralizing antibody to block respiratory disease and viral dissemination. Other models rely on the use of neonatal mice and less-physiological intracranial or intraperitoneal infection to induce mortality or paralysis similar to AFM (*39*, *42*–*44*). In a related set of manuscripts, mice were vaccinated with a B1 subclade–based VLP or inactivated virus, eliciting neutralizing antibodies against both the homologous strain and an A2 subclade virus (*32*, *33*, *39*). These studies showed a fourfold lower anti-B1 neutralizing response but twofold higher anti-A2 neutralizing response than was found in our hands. While these apparent differences are minor, they could be explained by selection of viral capsid sequences and/or differences in experimental procedures. While those studies demonstrated protection from lethal intraperitoneal challenge in the neonatal offspring of immunized mice or following passive transfer into newborn mice, we show the ability of vaccine-elicited neutralizing antibody to decrease B1 and B3 subclade virus infections in the lung and dissemination similar to that seen during human respiratory EV-D68 infections.

While the respiratory model enables assessment of antibody-mediated protection from mouse-adapted EV-D68 replication in the lung and dissemination to blood and spleen, it has limitations directly related to AFM and dissemination to the CNS [reviewed in (*42*)]. After intranasal infection, B1 subclade mouse-adapted EV-D68 (*38*) can be detected in the spinal cord and brain of 4-week-old AG129 mice; however, virus in the CNS does not cause paralysis after intranasal infection in AG129 mice unless they are infected at up to 5 days of age (*45*). After intranasal infection with our adapted B3 subclade EV-D68, we could detect virus in the spinal cord and brain of only one of six mice, and the quantity of virus recovered was at the limit of detection, suggesting that the B3 Mp20 virus may have a lower capacity than the B1 Mp30 virus to enter the CNS after intranasal infection (fig. S2D). Furthermore, we have observed no morbidity or mortality up to 4 weeks after intranasal infection of AG129 mice with the B3 mouse-adapted virus, further suggesting that the intranasal B3 Mp20/AG129 mouse model cannot be used to assess the EV-D68 neurological disease. The goal of this work is to demonstrate that the VLP vaccine–induced antibodies can prevent EV-D68 respiratory infections and dissemination into the blood; while these antibodies are assumed to block CNS entry, this must be experimentally confirmed using a different model.

It has been widely accepted for decades that the specificity of picornavirus-neutralizing antibody is determined by structural epitopes on viral particles (*46*, *47*). The basis for the observation that the B3 VLP (as compared to B1 VLP and B1 InVP) elicits higher levels of cross-neutralizing antibody against a virus in the A2 subclade is not clear. Using online tools such as Nextstrain and National Center for Biotechnology Information’s GenBank, analysis of the BC and DE loops in VP1 and the EF loop in VP2 demonstrates temporal antigenic variation in regions of the capsid that bind neutralizing antibodies (*6*, *48*). While there are amino acids in antigenic loops shared between the B3 and A2 subclade viruses used in this study that are consistent with neutralization data presented here, the predictive “hit” on neutralization capacity of any individual amino acid change in a VLP would need to be confirmed empirically.

We hypothesize that the elicitation of neutralizing antibodies that limit replication of EV-D68 in the respiratory tract will prevent neurological sequelae. The ability of systemic antibodies elicited by the IPV to prevent neurological disease despite virus replication in the gut is precedent for this notion (*49*). Epidemiologic evidence suggests that anti–EV-D68 neutralizing antibody titers greater than 1:256 are protective against severe and disseminated disease (*50*). On the basis of this prediction as well as our cross-subclade and cross-clade neutralization data, we anticipate that a B3-based vaccine would be a useful intervention for many years. Analysis of the amino acid sequences in the VP1 variable loops of 2021 and 2022 B clade viruses using the Nextstrain resource indicates that most isolates maintain the same BC and DE loop sequences as the US/2018-23209 VLP used in this study. As the B clade continues to evolve, studies using VLP based on the P1 sequences of future isolates could substantiate this prediction.

Despite multiple attempts, we were unable to mouse-adapt the A2 EV-D68 isolate US/KY/2014/18953, limiting our ability to evaluate protection against this subclade in vivo. Viruses in the A clade do cause substantial morbidity in humans, despite exhibiting different growth properties from B clade viruses in organ culture systems (*51*) and virulence in suckling mice (*39*). The evolving A2 subclade presents a risk to the elderly given the overall lower capacity of this population to neutralize A2 viruses (*5*, *52*, *53*). A vaccine capable of inducing protective neutralizing responses against currently circulating B3 and A2 subclade viruses would be ideal, and our NHP data indicate that a single B3 VLP may be sufficient to confer protection against both circulating subclades, representing an effective tool in the arsenal against future EV-D68 outbreaks. The difference we observed in cross-protection after vaccination of mice and NHP may be due to a combination of factors, including the inbred nature of the single strain of mice used to generate vaccine antisera, a specific activity of the Adjuplex adjuvant in macaques, or even a previous exposure to a macaque enterovirus. A larger study could shed light on the consistency and durability of cross-reactive responses in NHP. As EV-D68 continues to evolve to evade host immunity, the ease of modifying P1 capsid sequences to match those of emerging strains ensures flexibility to adapt and maximize vaccine efficacy. The VLP platform also readily enables multiplexing, and multivalent VLP vaccines may offer more comprehensive and durable protection against EV-D68 and allow for combination with VLP for other enteroviruses that are a threat to human health such as EV-A71.

The primary target population for an EV-D68 VLP vaccine is seronegative children. The average age of hospitalizations for EV-D68 severe respiratory disease and AFM is 4 to 6 years, depending on the outbreak year. We anticipate that the induction of balanced or T_H_1-polarized response would be favorable in young children, where T_H_2 responses to respiratory pathogens have been associated with exacerbation of asthma and eosinophilic inflammation (*54*, *55*). Alum adjuvants are T_H_2-polarizing (*56*, *57*), and because EV-D68 is a respiratory enterovirus with a primary site of infection in the lung, alternatives to alum could be used to elicit a more balanced T_H_ response. Adjuplex has been used in vaccine formulations for HIV (*58*) and influenza virus vaccines (*58*) and has been shown to elicit strong and balanced immune responses, including CD8^+^ T cell immunity (*59*, *60*). We confirm these findings here using IgG2a/c as a surrogate for T_H_1 induction. Unadjuvanted VLP and Alhydrogel formulations biased the antibody response toward IgG1 (T_H_2), while Adjuplex elicited a more balanced T_H_ response, particularly at the higher, typically used concentration (Fig. 4B and fig. S5). Future immunogenicity studies to assess T cell responses in mice and NHPs are warranted.

The future of EV-D68 outbreaks is unclear; AFM cases were down to 86% during the predicted biennial upsurge in 2020, yet in the late summer of 2022, pediatric intensive care units across the United States were overwhelmed with EV-D68 severe respiratory cases (*12*). These outbreaks put undue burden on pediatric health care facilities (*61*). Our data demonstrate that VLP based on B3 subclade EV-D68 potently elicit antibodies that protect mice from respiratory infection. These antibodies not only neutralize homologous B3 virus but also have a good cross-reactivity profile against heterologous A2 virus. As part of a pandemic preparedness plan for picornaviruses, the VLP vaccine platform embodies an amenable approach to rapidly respond as enterovirus strains emerge in the future.

## MATERIALS AND METHODS

### Viruses and cells

RD cells (American Type Culture Collection, CCL-136) were cultured in Dulbecco’s modified Eagle’s medium (DMEM) (Invitrogen) supplemented with 10% fetal bovine serum (Gemini Bioproducts) and penicillin/streptomycin (Invitrogen) in a cell culture incubator at 37°C. The viruses used in this study are listed in table S1. To make virus stocks, confluent monolayers of RD cells were infected at a multiplicity of infection (MOI) of 0.1 TCID_50_ per cell, incubated at 33°C in a cell culture incubator, and harvested when cells exhibited 95% cytopathic effect. The flask contents were frozen and thawed, clarified by low-speed centrifugation, and the supernatant was aliquoted and stored at −80°C. All stocks were titrated by infecting multiple wells of a 96-well plate containing RD cells with serial dilutions of the virus stock. The cytopathic effect on the wells was observed by fixing the cells with ExCellPlus fixative (StatLab) containing crystal violet. Virus quantitation of stocks was determined by TCID_50_ end point titration using the Spearman-Karber method (*62*).

### Generation of EV-D68 VLPs

Expression plasmids encoding for the P1 capsid or the 3CD protease polyproteins of EV-D68 isolate US/CO/14-93 were cotransfected into Expi293 suspension cells at a ratio of 4:1, respectively, using the ExpiFectamine 293 Transfection System (Thermo Fisher Scientific) following the manufacturer’s protocol. Four days after transfection, the cells were centrifuged at a low speed and resuspended in 1/10th the original culture volume with a TNE buffer [50 mM tris (pH 7.5), 150 mM NaCl, and 5 mM EDTA]. This resuspension was sonicated and clarified at 13,000*g* for 45 min. The supernatants were loaded on 20% sucrose cushions and centrifuged at 72,000*g* for 3 hours. The pellets were resuspended in 1.2 ml of TNE buffer, layered on 15 to 45% step sucrose gradients, and centrifuged at 220,000*g* for 3 hours. Fractions were collected from the bottom of the ultracentrifuge tube and tested for the presence of the formed VLP by dot blot using mAb228, an antibody against EV-D68. Dot blot–positive fractions were run on SDS-PAGE gels to detect the presence of viral capsid proteins by size estimation, and fractions with the highest concentrations of VLP were pooled, buffer-exchanged with 1× phosphate-buffered saline (PBS) on 100-kDa centrifugal filters (Amicon Ultra-15) and stored in aliquots at −80°C. Purified VLP was electrophoresed on SDS-PAGE to verify protein content and then confirmed to contain VP1 by Western blot with anti–EV-D68 VP1 (GeneTex, #132312) antibody. Purified VLP were used as an antigen in a binding ELISA with the EV-D68 mAb228 and mAb219 and visualized by negative-stain transmission electron microscopy.

### Generation of inactivated EV-D68 virions

Confluent monolayers of RD cells were infected with EV-D68 B1 subclade isolate US/MO/2014-18947 at an MOI of 0.05 TCID_50_ per cell. When the infection reached 95% cytopathic effect, the cells and media were collected and subjected to three freeze/thaw cycles. PEG-8000 (polyethylene glycol, molecular weight 800; Sigma-Aldrich) was added to the clarified lysate at a final concentration of 8% (w/v) and stirred for 16 hours at 4°C. The PEG mixture was centrifuged at 13,000*g*, and the pellet was resuspended in 1/10th the original volume with TNM buffer (10 mM tris-HCI, 200 mM NaCI, and 50 mM MgCI_2_). This resuspension was layered on 30% sucrose cushions and centrifuged at 210,000*g* for 3 hours. The sucrose cushion pellets were pooled and subjected to an additional 30% sucrose cushion centrifugation. This single pellet was resuspended in 1 ml of TNE buffer, layered on a 15 to 50% sucrose gradient and centrifuged at 220,000*g* for 3 hours. Fractions were collected from the bottom and analyzed for protein content by bicinchoninic acid (Pierce) and RNA by spectrophotometry (NanoDrop). Fractions containing high-protein and RNA concentrations were separated by SDS-PAGE to detect the presence of viral capsid proteins by size estimation (Fig. 1B) and then confirmed to contain VP1 by Western blot with an anti–EV-D68 VP1 antibody (GeneTex, #132312; Fig. 1C), respectively. Fractions with the highest concentrations of virus were pooled and buffer-exchanged to 1× PBS on 100-kDa spin tubes (Amicon Ultra-15). To inactivate the virus, β-propiolactone (MilliporeSigma, P5648) was added at a ratio of 1:4000 (v/v) and incubated at 4°C for 18 hours. The preparation was shifted to 37°C for 1 hour to hydrolyze any remaining β-propiolactone, and then aliquots were frozen at −80°C. The inactivation of the virus particles was confirmed by serial blind passage on RD cells. The resulting inactivated virions were used as antigen in a binding ELISA with the EV-D68 mAb228 and mAb219 and visualized by negative-stain transmission electron microscopy.

### Vaccination of mice

All animal procedures had prior approval from the National Institutes of Health (NIH) Vaccine Research Center Animal Care and Use Committee, protocols VRC-18-0773, VRC-19-0823, and VRC-21-0920. B1 subclade VLP and inactivated virus preparations were formulated with SAS, and 8-week-old CB6F1 mice (Jackson Laboratory) were immunized intramuscularly with a half dose in each inner thigh. Four weeks later, mice were boosted with the same immunogen. Two weeks after each vaccination, individual serum samples were obtained from each mouse and stored at −20°C until further use. Terminal bleeds were obtained 12 weeks after prime vaccination. For B3 subclade VLP experiments, CB6F1 mice were vaccinated with unadjuvanted VLP, SAS, Alhydrogel, or Adjuplex VLP formulations in a prime/boost regimen similar to that of the B1 immunogens, and the terminal bleed was taken 18 weeks after prime vaccination.

### Vaccination of NHPs

The macaque study had prior approval by the Vaccine Research Center Animal Care and Use Committee, protocol VRC-21-0933. Two rhesus macaques, one female and one male, were immunized intramuscularly with 50 μg of B3 VLP formulated with 20% Adjuplex with a half dose in each leg muscle. Four weeks later, both animals were boosted with the same vaccine. Sera were obtained on the schedule described in the schema in Fig. 6B. All animal handling procedures were done by members of the NIH NHP Immunogenicity Core.

### Neutralization assay

Serum samples were heat-inactivated at 56°C for 1 hour and then diluted in DMEM to make twofold serial dilutions in wells of a nontissue culture–treated 96-well dilution plate. An equal volume of DMEM containing 100 TCID_50_ of virus was added to each well containing serum dilutions and incubated at 33°C in a cell culture incubator. After 1 hour, the serum/virus mixtures were added to wells containing 95% confluent monolayers of RD cells and incubated at 33°C in a cell culture incubator. After 1 hour, DMEM supplemented with 1% fetal bovine serum and antibiotics was added to each well, and the plates were incubated for 5 days at 33°C in a cell culture incubator. After 5 days, the cells were scored for the presence of cytopathic effects and fixed. The end point neutralization titer is defined as the highest serum dilution that blocks the development of any viral CPE. Each serum sample was tested in a minimum of two separate assays with two technical replicates in each assay.

### IgG subclass ELISA

MaxiSorp plates (Thermo Fisher Scientific) were coated with EV-D68 B3 VLP (2 μg/ml) in PBS overnight at 4°C. The following day, the plates were washed three times with 300 μl per well of PBS containing 0.05% (v/v) Tween 20 and blocked for 2 hours at room temperature with 200 μl per well of PBS containing 0.05% (v/v) Tween 20 and 1% (w/v) nonfat dry milk (blocking buffer). The plates were washed again as described, and mouse sera were serially diluted fourfold with a starting dilution of 1:100 in blocking buffer. One hundred microliters of diluted samples was applied to each well and plates were incubated at room temperature for 1 hour. Plates were washed as described and incubated with a 1:5000 dilution of goat anti-mouse IgG_1_ human ads–horseradish peroxidase (HRP) (SouthernBiotech, #1070-05) or goat anti-mouse IgG_2A_ human ads-HRP (SouthernBiotech, #1080-05) and goat anti-mouse IgG_2c_ human ads-HRP (SouthernBiotech, #1079-05) in blocking buffer for 1 hour at room temperature. Plates were washed as described and developed with 100 μl per well of KPL SureBlue 1-component TMB microwell peroxidase substrate at room temperature for 10 min. Development was stopped by addition of 100 μl per well of 1-N sulfuric acid, and absorbance was immediately read at 450 and 650 nm. For analysis, absorbance at 650 nm was subtracted from the absorbance at 450 nm. Serum dilutions were log-transformed, and absorbance values were fit by nonlinear regression using GraphPad Prism software. End point titer was determined as the dilution resulting in absorbance values fourfold above the average of negative control wells. Mean end point titers were compared by one-way analysis of variance (ANOVA) and Sidak’s test for multiple comparisons as only IgG_1_ and IgG_2_ end point titers for each adjuvant condition were compared to each other.

### Infection of mice and detection of virus in mouse tissues

Three- to four-week-old AG129 mice (IFNαβγR^−/−^; Marshall BioResources) were anesthetized with isoflurane and intranasally infected by pipetting 50 μl of undiluted mouse-adapted EV-D68 into the nostrils while held upright. The input dose was confirmed by titrating the remaining inoculum after the intranasal infections were completed. To detect virus in vivo, mice were euthanized with sodium pentobarbital, and blood, spleen, and the left lobe of the lung were extracted from the mice and then frozen on dry ice. Blood was processed by clotting at room temperature for 30 min, and serum was separated by centrifugation at 3000*g*. Serum samples were stored at −80°C until titration. Lung and spleen tissues were processed by adding 2 ml of DMEM supplemented with 1% fetal bovine serum and antibiotics to each tube containing frozen tissue and then transferred to a gentleMACS M (Miltenyi Biotec Inc.) tube. Tissues were homogenized using the Protein.01 program of the gentleMACS dissociator (Miltenyi Biotec Inc.) and centrifuged at 3000*g* for 10 min. These clarified and homogenized tissues and serum samples were titrated on RD cells as described above and stored at −80°C. Samples negative for virus were confirmed by quantitative reverse transcription polymerase chain reaction using pan-enterovirus 2A gene-specific primers and probe (forward: TGTCCACATGGGGTAATYGG, reverse: TGCCTTGTTCCATAGCATCAGT, probe: ACAGCAGGRGGGGGTGGAATTGT).

### Purification and passive transfer of IgG

IgG from serum samples collected from terminal bleeds of B1 immunogen–vaccinated CB6F1 mice were purified by binding to a protein G Sepharose resin (GE Healthcare) and then the IgG was eluted according to the manufacturer’s protocol as previously described (*63*); IgG from serum samples collected from terminal bleeds of B3-VLP–vaccinated CB6F1 mice were purified using protein A agarose (Pierce) according to the manufacturer’s protocol. IgG was concentrated by centrifugal filtration (Amicon Ultra, EMD Millipore), diluted in PBS and injected interperitoneally into AG129 mice. The next day, serum samples were taken from the mice to determine the neutralization titer before intranasal challenge with mouse-adapted EV-D68.

### Adaptation of EV-D68 B3 subclade to AG129 mice and preparation of challenge stocks

Two 4-week-old AG129 mice were infected intranasally with 10^5^ TCID_50_ EV-D68 US/2018-23087. Two days after infection, lungs were harvested for virus isolation as described above. The lung homogenates were serially passaged via intranasal infection through mouse lungs (*n* = 2 mice per passage) eight times. The passage 8 lung homogenates were expanded by infection of RD cells. The resulting RD virus stock was used to infect 12 AG129 mice to make a lung homogenate preparation from pooled lungs harvested 2 days after infection. The pool, designated as EV-D68 2018-23087 Mp9, was aliquoted; stored at −80°C; and was used in the passive transfer/challenge study described in Fig. 3B. To further adapt the virus, the Mp9 virus was serially passaged through AG129 mouse lungs for an additional 10 times. The passage 19 lung homogenate pool was expanded by infection of RD cells. This RD virus stock was used to infect 10 AG129 mice to make a lung homogenate preparation from pooled lungs harvested 2 days after infection. The pool, designated as EV-D68 2018-23087 Mp20, was aliquoted and stored at −80°C and was used in the passive transfer/challenge study described in Fig. 5A.

### Statistical analysis

Statistics were performed using GraphPad Prism (version 9.3.1). End point antibody neutralization titers or virus titers from lung tissues were log-transformed and then tested for normality before ANOVA as described in the figure legends. *P* values indicate significance as follows: *P < 0.05, **P < 0.01, ***P < 0.001, and ****P < 0.0001. Pearson correlations were performed between log_10_-transformed virus titers in mouse lung tissue and log_2_-transformed post-antibody transfer neutralizing antibody titers.

## Notes

View/request a protocol for this paper from *Bio-protocol*.

## References

[1] J. H. Schieble, V. L. Fox, E. H. Lennette, A probable new human picornavirus associated with respiratory diseases. Am. J. Epidemiol. 85, 297–310 (1967). 4960233 10.1093/oxfordjournals.aje.a120693

[2] G. Wang, J. Zhuge, W. Huang, S. M. Nolan, V. L. Gilrane, C. Yin, N. Dimitrova, J. T. Fallon, Enterovirus D68 subclade B3 strain circulating and causing an outbreak in the United States in 2016. Sci. Rep. 7, 1242 (2017). 28455514 10.1038/s41598-017-01349-4 PMC5430842

[3] A. Lopez, A. Lee, A. Guo, J. L. Konopka-Anstadt, A. Nisler, S. L. Rogers, B. Emery, W. A. Nix, S. Oberste, J. Routh, M. Patel, Vital signs: Surveillance for acute flaccid myelitis—United States, 2018. MMWR Morb. Mortal. Wkly Rep. 68, 608–614 (2019). 31295232 10.15585/mmwr.mm6827e1

[4] V. L. Gilrane, J. Zhuge, W. Huang, S. M. Nolan, A. Dhand, C. Yin, C. Salib, F. Shakil, H. Engel, J. T. Fallon, G. Wang, Biennial upsurge and molecular epidemiology of enterovirus D68 infection in New York, USA, 2014 to 2018. J. Clin. Microbiol. 58, e00284-20 (2020). 32493783 10.1128/JCM.00284-20 PMC7448634

[5] M. Duval, A. Mirand, O. Lesens, J. O. Bay, D. Caillaud, D. Gallot, A. Lautrette, S. Montcouquiol, J. Schmidt, C. Egron, G. Jugie, M. Bisseux, C. Archimbaud, C. Lambert, C. Henquell, J.-L. Bailly, Retrospective study of the upsurge of enterovirus D68 clade D1 among adults (2014-2018). Viruses 13, 1607 (2021). 34452471 10.3390/v13081607 PMC8402803

[6] E. B. Hodcroft, R. Dyrdak, C. Andres, A. Egli, J. Reist, D. G. M. de Artola, J. Alcoba-Florez, H. G. M. Niesters, A. Anton, R. Poelman, M. Reynders, E. Wollants, R. A. Neher, J. Albert, Evolution, geographic spreading, and demographic distribution of Enterovirus D68. PLOS Pathog. 18, e1010515 (2022). 35639811 10.1371/journal.ppat.1010515 PMC9212145

[7] O. C. Murphy, C. A. Pardo, Acute flaccid myelitis: A clinical review. Semin. Neurol. 40, 211–218 (2020). 32143233 10.1055/s-0040-1705123

[8] M. R. Vogt, P. F. Wright, W. F. Hickey, T. De Buysscher, K. L. Boyd, J. E. Crowe Jr., Enterovirus D68 in the anterior horn cells of a child with acute flaccid myelitis. N. Engl. J. Med. 386, 2059–2060 (2022). 35613028 10.1056/NEJMc2118155 PMC9321432

[9] S. W. Park, M. Pons-Salort, K. Messacar, C. Cook, L. Meyers, J. Farrar, B. T. Grenfell, Epidemiological dynamics of enterovirus D68 in the United States and implications for acute flaccid myelitis. Sci. Transl. Med. 13, 10.1126/scitranslmed.abd2400, (2021). 10.1126/scitranslmed.abd2400 33692131

[10] A. Fall, N. Gallagher, C. P. Morris, J. M. Norton, A. Pekosz, E. Klein, H. H. Mostafa, Circulation of enterovirus D68 during period of increased influenza-like illness, Maryland, USA, 2021. Emerg. Infect. Dis. 28, 1525–1527 (2022). 35642471 10.3201/eid2807.212603 PMC9239864

[11] K. S. Benschop, J. Albert, A. Anton, C. Andres, M. Aranzamendi, B. Armannsdottir, J.-L. Bailly, F. Baldanti, G. E. Baldvinsdottir, S. Beard, N. Berginc, S. Bottcher, S. Blomqvist, L. Bubba, C. Calvo, M. Cabrerizo, A. Cavallero, C. Celma, F. Ceriotti, I. Costa, S. Cottrell, M. Del Cuerpo, J. Dean, J. L. Dembinski, S. Diedrich, J. Diez-Domingo, D. Dorenberg, E. Duizer, R. Dyrdak, D. Fanti, A. Farkas, S. Feeney, J. Flipse, C. De Gascun, C. Galli, I. Georgieva, L. Gifford, R. Guiomar, M. Honemann, N. Ikonen, M. Jeannoel, L. Josset, K. Keeren, F. X. Lopez-Labrador, M. Maier, J. McKenna, A. Meijer, B. Mengual-Chulia, S. E. Midgley, A. Mirand, M. Montes, C. Moore, U. Morley, J. L. Murk, L. Nikolaeva-Glomb, S. Numanovic, M. Oggioni, P. Palminha, E. Pariani, L. Pellegrinelli, A. Piralla, C. Pietsch, L. Pineiro, N. Rabella, P. Rainetova, S. C. U. Renteria, M. P. Romero, M. Reynders, L. Roorda, C. Savolainen-Kopra, I. Schuffenecker, A. Soynova, C. M. Swanink, T. Ursic, J. J. Verweij, J. Vila, T. Vuorinen, P. Simmonds, T. K. Fischer, H. Harvala, Re-emergence of enterovirus D68 in Europe after easing the COVID-19 lockdown, September 2021. Euro Surveill. 26, 2100998 (2021). 34763750 10.2807/1560-7917.ES.2021.26.45.2100998 PMC8646978

[12] K. C. Ma, A. Winn, H. L. Moline, H. M. Scobie, C. M. Midgley, H. L. Kirking, J. Adjemian, K. P. Hartnett, D. Johns, J. M. Jones, A. Lopez, X. Lu, A. Perez, C. G. Perrine, A. E. Rzucidlo, M. L. McMorrow, B. J. Silk, Z. Stein, E. Vega; New Vaccine Surveillance Network Collaborators, A. J. Hall, Increase in acute respiratory illnesses among children and adolescents associated with rhinoviruses and enteroviruses, including enterovirus D68—United States, July-September 2022. MMWR Morb. Mortal. Wkly Rep. 71, 1265–1270 (2022). 36201400 10.15585/mmwr.mm7140e1 PMC9541033

[13] A. Christy, K. Messacar, Acute flaccid myelitis associated with enterovirus D68: A review. J. Child Neurol. 34, 511–516 (2019). 31014167 10.1177/0883073819838376

[14] National Center for Immunization and Respiratory Diseases, “Acute flaccid myelitis (AFM): Clinical guidance for the acute medical treatment of AFM” (2022); www.cdc.gov/acute-flaccid-myelitis/hcp/clinical-management.html [accesed 14 March 2023].

[15] M. J. Rudy, J. Frost, P. Clarke, K. L. Tyler, Neutralizing antibody given after paralysis onset reduces the severity of paralysis compared to nonspecific antibody-treated controls in a mouse model of EV-D68-associated acute flaccid myelitis. Antimicrob. Agents Chemother. 66, e0022722 (2022). 35894595 10.1128/aac.00227-22 PMC9380545

[16] M. R. Vogt, J. Fu, N. Kose, L. E. Williamson, R. Bombardi, I. Setliff, I. S. Georgiev, T. Klose, M. G. Rossmann, Y. A. Bochkov, J. E. Gern, R. J. Kuhn, J. E. Crowe Jr., Human antibodies neutralize enterovirus D68 and protect against infection and paralytic disease. Sci. Immunol. 5, eaba4902 (2020). 32620559 10.1126/sciimmunol.aba4902 PMC7418079

[17] C. Zhang, C. Xu, W. Dai, Y. Wang, Z. Liu, X. Zhang, X. Wang, H. Wang, S. Gong, Y. Cong, Z. Huang, Functional and structural characterization of a two-MAb cocktail for delayed treatment of enterovirus D68 infections. Nat. Commun. 12, 2904 (2021). 34006855 10.1038/s41467-021-23199-5 PMC8131599

[18] National Center for Immunization and Respiratory Diseases, “Acute flaccid myelitis: Clinicians and health departments” (2022); www.cdc.gov/acute-flaccid-myelitis/hcp/clinicians-health-departments.html [accessed 14 March 2023].

[19] D. M. Horstmann, Control of poliomyelitis: A continuing paradox. J. Infect. Dis. 146, 540–551 (1982). 6288809 10.1093/infdis/146.4.540

[20] K. Chumakov, E. Ehrenfeld, V. I. Agol, E. Wimmer, Polio eradication at the crossroads. Lancet Glob. Health 9, e1172–e1175 (2021). 34118192 10.1016/S2214-109X(21)00205-9

[21] M. T. Yeh, E. Bujaki, P. T. Dolan, M. Smith, R. Wahid, J. Konz, A. J. Weiner, A. S. Bandyopadhyay, P. Van Damme, I. De Coster, H. Revets, A. Macadam, R. Andino, Engineering the live-attenuated polio vaccine to prevent reversion to virulence. Cell Host Microbe 27, 736–751.e8 (2020). 32330425 10.1016/j.chom.2020.04.003 PMC7566161

[22] F. Diaz-San Segundo, G. N. Medina, C. Stenfeldt, J. Arzt, T. de Los Santos, Foot-and-mouth disease vaccines. Vet. Microbiol. 206, 102–112 (2017). 28040311 10.1016/j.vetmic.2016.12.018

[23] M. R. Vogt, J. E. Crowe Jr., Current understanding of humoral immunity to enterovirus D68. J. Pediatric Infect. Dis. Soc. 7, S49–S53 (2018). 30590621 10.1093/jpids/piy124

[24] J. M. Hogle, M. Chow, D. J. Filman, Three-dimensional structure of poliovirus at 2.9 A resolution. Science 229, 1358–1365 (1985). 2994218 10.1126/science.2994218

[25] M. G. Murray, R. J. Kuhn, M. Arita, N. Kawamura, A. Nomoto, E. Wimmer, Poliovirus type 1/type 3 antigenic hybrid virus constructed in vitro elicits type 1 and type 3 neutralizing antibodies in rabbits and monkeys. Proc. Natl. Acad. Sci. U.S.A. 85, 3203–3207 (1988). 2834736 10.1073/pnas.85.9.3203 PMC280172

[26] A. Meijer, S. van der Sanden, B. E. P. Snijders, G. Jaramillo-Gutierrez, L. Bont, C. K. van der Ent, P. Overduin, S. L. Jenny, E. Jusic, H. G. A. M. van der Avoort, G. J. D. Smith, G. A. Donker, M. P. G. Koopmans, Emergence and epidemic occurrence of enterovirus 68 respiratory infections in The Netherlands in 2010. Virology 423, 49–57 (2012). 22177700 10.1016/j.virol.2011.11.021

[27] E. B. Hodcroft, R. A. Neher, R. Dyrdak, J. Albert, “Phylodynamics of enterovirus D68” (2022); https://nextstrain.org/enterovirus/d68/genome [accessed 14 March 2023].

[28] P.-Y. Lim, A. C. Hickey, M. F. Jamiluddin, S. Hamid, J. Kramer, R. Santos, K. N. Bossart, M. J. Cardosa, Immunogenicity and performance of an enterovirus 71 virus-like-particle vaccine in nonhuman primates. Vaccine 33, 6017–6024 (2015). 26271825 10.1016/j.vaccine.2015.05.108

[29] L. Sherry, K. Grehan, J. S. Snowden, M. L. Knight, O. O. Adeyemi, D. J. Rowlands, N. J. Stonehouse, Comparative molecular biology approaches for the production of poliovirus virus-like particles using *Pichia pastoris*. mSphere 5, e00838-19 (2020). 32161150 10.1128/mSphere.00838-19 PMC7067596

[30] M. W. Bahar, C. Porta, H. Fox, A. J. Macadam, E. E. Fry, D. I. Stuart, Mammalian expression of virus-like particles as a proof of principle for next generation polio vaccines. NPJ Vaccines 6, 5 (2021). 33420068 10.1038/s41541-020-00267-3 PMC7794334

[31] M. Puckette, V. Primavera, E. Martel, J. Barrera, W. Hurtle, B. Clark, B. Kamicker, M. Zurita, D. Brake, J. Neilan, Transiently transfected mammalian cell cultures: An adaptable and effective platform for virus-like particle-based vaccines against foot-and-mouth disease virus. Viruses 14, 989 (2022). 35632734 10.3390/v14050989 PMC9147724

[32] W. Dai, C. Zhang, X. Zhang, P. Xiong, Q. Liu, S. Gong, L. Geng, D. Zhou, Z. Huang, A virus-like particle vaccine confers protection against enterovirus D68 lethal challenge in mice. Vaccine 36, 653–659 (2018). 29295756 10.1016/j.vaccine.2017.12.057

[33] C. Zhang, X. Zhang, W. Zhang, W. Dai, J. Xie, L. Ye, H. Wang, H. Chen, Q. Liu, S. Gong, L. Geng, Z. Huang, Enterovirus D68 virus-like particles expressed in *Pichia pastoris* potently induce neutralizing antibody responses and confer protection against lethal viral infection in mice. Emerg. Microbes Infect. 7, 3 (2018). 29323105 10.1038/s41426-017-0005-x PMC5837163

[34] H. Dong, H.-C. Guo, S.-Q. Sun, Virus-like particles in picornavirus vaccine development. Appl. Microbiol. Biotechnol. 98, 4321–4329 (2014). 24647496 10.1007/s00253-014-5639-1

[35] A. M. King, B. O. Underwood, D. McCahon, J. W. Newman, F. Brown, Biochemical identification of viruses causing the 1981 outbreaks of foot and mouth disease in the UK. Nature 293, 479–480 (1981). 6273731 10.1038/293479a0

[36] F. Brown, Review of accidents caused by incomplete inactivation of viruses. Dev. Biol. Stand. 81, 103–107 (1993). 8174792

[37] B. A. Phillips, In vitro assembly of poliovirus. II. Evidence for the self-assembly of 14 S particles into empty capsids. Virology 44, 307–316 (1971). 4105254 10.1016/0042-6822(71)90262-5

[38] W. J. Evans, B. L. Hurst, C. J. Peterson, A. J. Van Wettere, C. W. Day, D. F. Smee, E. B. Tarbet, Development of a respiratory disease model for enterovirus D68 in 4-week-old mice for evaluation of antiviral therapies. Antiviral Res. 162, 61–70 (2019). 30521834 10.1016/j.antiviral.2018.11.012 PMC6997929

[39] C. Zhang, X. Zhang, W. Dai, Q. Liu, P. Xiong, S. Wang, L. Geng, S. Gong, Z. Huang, A mouse model of enterovirus D68 infection for assessment of the efficacy of inactivated vaccine. Viruses 10, 58 (2018). 29385753 10.3390/v10020058 PMC5850365

[40] C. C. Holm-Hansen, S. E. Midgley, T. K. Fischer, Global emergence of enterovirus D68: A systematic review. Lancet Infect. Dis. 16, e64–e75 (2016). 26929196 10.1016/S1473-3099(15)00543-5

[41] H. C. Howson-Wells, T. Tsoleridis, I. Zainuddin, A. W. Tarr, W. L. Irving, J. K. Ball, L. Berry, G. Clark, C. P. McClure, Enterovirus D68 epidemic, UK, 2018, was caused by subclades B3 and D1, predominantly in children and adults, respectively, with both subclades exhibiting extensive genetic diversity. Microb. Genom. 8, mgen000825 (2022). 35532121 10.1099/mgen.0.000825 PMC9465064

[42] M. S. Vermillion, J. Dearing, Y. Zhang, D. R. Adney, R. H. Scheuermann, A. Pekosz, E. B. Tarbet, Animal models of enterovirus D68 infection and disease. J. Virol. 96, e0083322 (2022). 35852353 10.1128/jvi.00833-22 PMC9364802

[43] Q. Zheng, R. Zhu, L. Xu, M. He, X. Yan, D. Liu, Z. Yin, Y. Wu, Y. Li, L. Yang, W. Hou, S. Li, Z. Li, Z. Chen, Z. Li, H. Yu, Y. Gu, J. Zhang, T. S. Baker, Z. H. Zhou, B. S. Graham, T. Cheng, S. Li, N. Xia, Atomic structures of enterovirus D68 in complex with two monoclonal antibodies define distinct mechanisms of viral neutralization. Nat. Microbiol. 4, 124–133 (2019). 30397341 10.1038/s41564-018-0275-7 PMC6727974

[44] A. M. Hixon, G. Yu, J. S. Leser, S. Yagi, P. Clarke, C. Y. Chiu, K. L. Tyler, A mouse model of paralytic myelitis caused by enterovirus D68. PLOS Pathog. 13, e1006199 (2017). 28231269 10.1371/journal.ppat.1006199 PMC5322875

[45] J. D. Morrey, H. Wang, B. L. Hurst, K. Zukor, V. Siddharthan, A. J. Van Wettere, D. G. Sinex, E. B. Tarbet, Causation of acute flaccid paralysis by myelitis and myositis in enterovirus-D68 infected mice deficient in interferon αβ/γ receptor deficient mice. Viruses 10, 33 (2018). 29329211 10.3390/v10010033 PMC5795446

[46] M. G. Rossmann, The canyon hypothesis. J. Biol. Chem. 264, 14587–14590 (1989). 2670920

[47] P. D. Minor, Antigenic structure of picornaviruses. Curr. Top. Microbiol. Immunol. 161, 121–154 (1990). 2169382 10.1007/978-3-642-75602-3_5

[48] Y. Fang, Q. Chen, H. Wang, L. Wang, H. Rong, Q. Liao, C. Dong, The role of conformational epitopes in the evolutionary divergence of enterovirus D68 clades: A bioinformatics-based study. Infect. Genet. Evol. 93, 104992 (2021). 34242773 10.1016/j.meegid.2021.104992

[49] E. P. Parker, N. A. Molodecky, M. Pons-Salort, K. M. O'Reilly, N. C. Grassly, Impact of inactivated poliovirus vaccine on mucosal immunity: Implications for the polio eradication endgame. Expert Rev. Vaccines 14, 1113–1123 (2015). 26159938 10.1586/14760584.2015.1052800 PMC4673562

[50] R. A. Livingston, C. J. Harrison, R. Selvarangan, Neutralizing enterovirus D68 antibodies in children after 2014 outbreak, Kansas City, Missouri, USA. Emerg. Infect. Dis. 28, 539–547 (2022). 35201738 10.3201/eid2803.211467 PMC8888215

[51] M. C. Freeman, A. I. Wells, J. Ciomperlik-Patton, M. M. Myerburg, L. Yang, J. Konopka-Anstadt, C. B. Coyne, Respiratory and intestinal epithelial cells exhibit differential susceptibility and innate immune responses to contemporary EV-D68 isolates. eLife 10, e66687 (2021). 34196272 10.7554/eLife.66687 PMC8285104

[52] S. K. P. Lau, C. C. Y. Yip, P. S.-H. Zhao, W.-N. Chow, K. K. W. To, A. K. L. Wu, K.-Y. Yuen, P. C. Y. Woo, Enterovirus D68 infections associated with severe respiratory illness in elderly patients and emergence of a novel clade in Hong Kong. Sci. Rep. 6, 25147 (2016). 27121085 10.1038/srep25147 PMC4848506

[53] C. J. Harrison, W. C. Weldon, B. A. Pahud, M. A. Jackson, M. S. Oberste, R. Selvarangan, Neutralizing antibody against enterovirus D68 in children and adults before 2014 outbreak, Kansas City, Missouri, USA. Emerg. Infect. Dis. 25, 585–588 (2019). 30789123 10.3201/eid2503.180960 PMC6390745

[54] A. S. Price, J. L. Kennedy, T-helper 2 mechanisms involved in human rhinovirus infections and asthma. Ann. Allergy Asthma Immunol. 129, 681–691 (2022). 36002092 10.1016/j.anai.2022.08.015 PMC10316285

[55] T. Jartti, K. Bonnelykke, V. Elenius, W. Feleszko, Role of viruses in asthma. Semin. Immunopathol. 42, 61–74 (2020). 31989228 10.1007/s00281-020-00781-5 PMC7066101

[56] L. Bungener, F. Geeraedts, W. Ter Veer, J. Medema, J. Wilschut, A. Huckriede, Alum boosts TH2-type antibody responses to whole-inactivated virus influenza vaccine in mice but does not confer superior protection. Vaccine 26, 2350–2359 (2008). 18400340 10.1016/j.vaccine.2008.02.063

[57] K. S. Korsholm, R. V. Petersen, E. M. Agger, P. Andersen, T-helper 1 and T-helper 2 adjuvants induce distinct differences in the magnitude, quality and kinetics of the early inflammatory response at the site of injection. Immunology 129, 75–86 (2010). 19824919 10.1111/j.1365-2567.2009.03164.x PMC2807488

[58] F. Wegmann, A. E. Moghaddam, T. Schiffner, K. H. Gartlan, T. J. Powell, R. A. Russell, M. Baart, E. W. Carrow, Q. J. Sattentau, The carbomer-lecithin adjuvant adjuplex has potent immunoactivating properties and elicits protective adaptive immunity against influenza virus challenge in mice. Clin. Vaccine Immunol. 22, 1004–1012 (2015). 26135973 10.1128/CVI.00736-14 PMC4550664

[59] D. J. Gasper, B. Neldner, E. H. Plisch, H. Rustom, E. Carrow, H. Imai, Y. Kawaoka, M. Suresh, Effective respiratory CD8 T-cell immunity to influenza virus induced by intranasal carbomer-lecithin-adjuvanted non-replicating vaccines. PLOS Pathog. 12, e1006064 (2016). 27997610 10.1371/journal.ppat.1006064 PMC5173246

[60] W. Lee, B. Kingstad-Bakke, B. Paulson, A. Larsen, K. Overmyer, C. B. Marinaik, K. Dulli, R. Toy, G. Vogel, K. P. Mueller, K. Tweed, A. J. Walsh, J. Russell, K. Saha, L. Reyes, M. C. Skala, J. D. Sauer, D. M. Shayakhmetov, J. Coon, K. Roy, M. Suresh, Carbomer-based adjuvant elicits CD8 T-cell immunity by inducing a distinct metabolic state in cross-presenting dendritic cells. PLOS Pathog. 17, e1009168 (2021). 33444400 10.1371/journal.ppat.1009168 PMC7840022

[61] K. Messacar, S. M. Hawkins, J. Baker, K. Pearce, S. Tong, S. R. Dominguez, S. Parker, Resource burden during the 2014 enterovirus D68 respiratory disease outbreak at children's hospital Colorado: An unexpected strain. JAMA Pediatr. 170, 294–297 (2016). 26783716 10.1001/jamapediatrics.2015.3879

[62] J. Hierholzer, R. Killington, Virus isolation and quantitation, in *Virology Methods Manual,* B. W. J. Mahy, H. O. Kangro, Eds. (Academic Press, 1996), pp. 25–46.

[63] S. Maciejewski, T. J. Ruckwardt, K. M. Morabito, B. M. Foreman, K. E. Burgomaster, D. N. Gordon, R. S. Pelc, C. R. DeMaso, S.-Y. Ko, B. E. Fisher, E. S. Yang, D. Nair, K. E. Foulds, J. P. Todd, W. P. Kong, V. Roy, M. Aleshnick, S. D. Speer, N. Bourne, A. D. Barrett, M. C. Nason, M. Roederer, M. R. Gaudinski, G. L. Chen, K. A. Dowd, J. E. Ledgerwood, G. Alter, J. R. Mascola, B. S. Graham, T. C. Pierson, Distinct neutralizing antibody correlates of protection among related Zika virus vaccines identify a role for antibody quality. Sci. Transl. Med. 12, eaaw9066 (2020). 32522807 10.1126/scitranslmed.aaw9066

