# Supplementary material for: EV-D68 virus-like particle vaccines elicit cross-clade neutralizing antibodies that inhibit infection and block dissemination
Source: Sci Adv. 2023 May 17;9(20):eadg6076. doi: 10.1126/sciadv.adg6076 (PMC10191429; doi:10.1126/sciadv.adg6076)
Supplement: Supplementary file 1 — Figs. S1 to S6 Tables S1 and S2 [file sciadv.adg6076_sm.pdf]

Science Advances
AAAS

Supplementary Materials for

EV-D68 virus-like particle vaccines elicit cross-clade neutralizing antibodies that inhibit infection and block dissemination

Peter W. Krug *et al.*

Corresponding author: Peter W. Krug, peter.krug@nih.gov; Tracy J. Ruckwardt, truckwardt@mail.nih.gov

Sci. Adv. **9**, eadg6076 (2023)
DOI: 10.1126/sciadv.adg6076

This PDF file includes:

Figs. S1 to S6
Tables S1 and S2

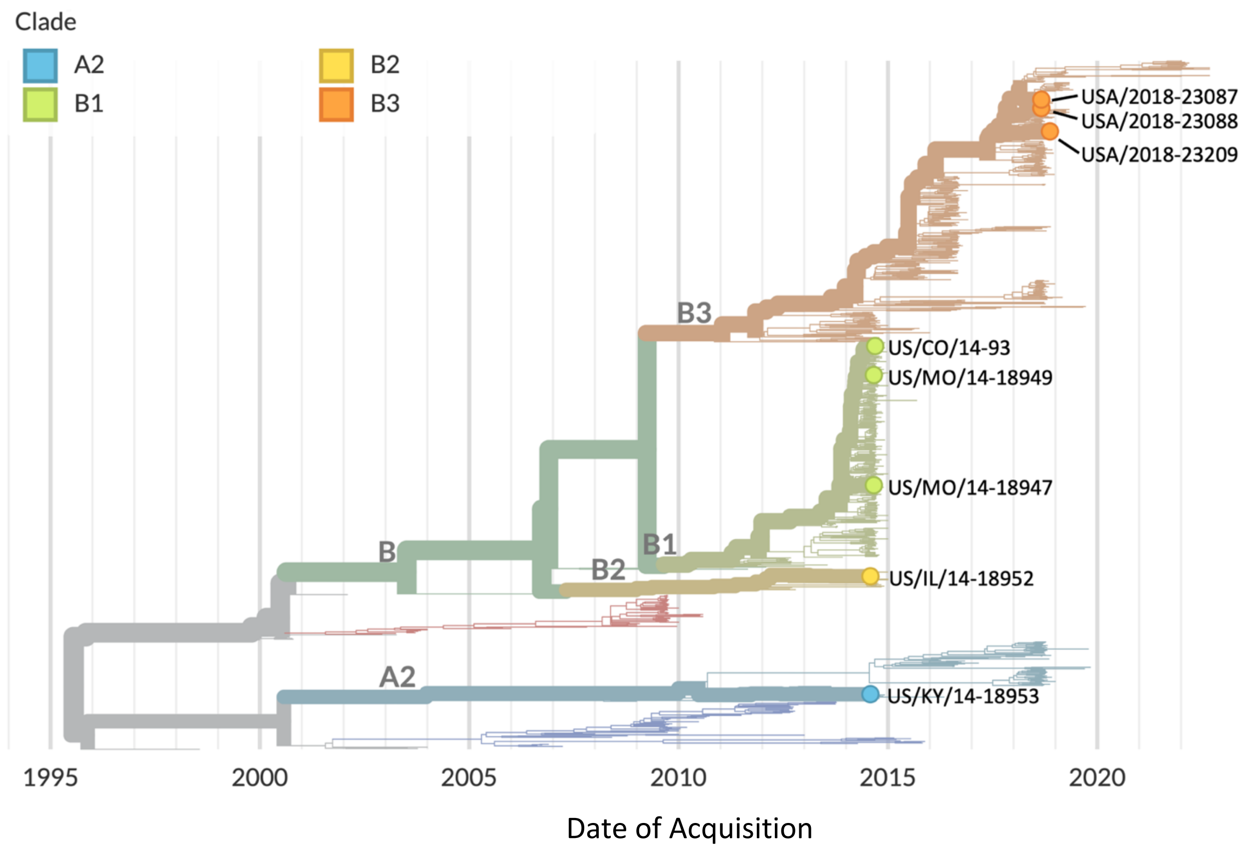

Figure S1. EV-D68 phylogeny highlighting the viruses and viral nucleic acid sequences used in this study. The A1 and C clades were not included for simplicity. Reproduced from: https://nextstrain.org/enterovirus/d68/genome?c=clade_membership as accessed on 03/08/2023. Screenshot used under a CC-BY-4.0 license.

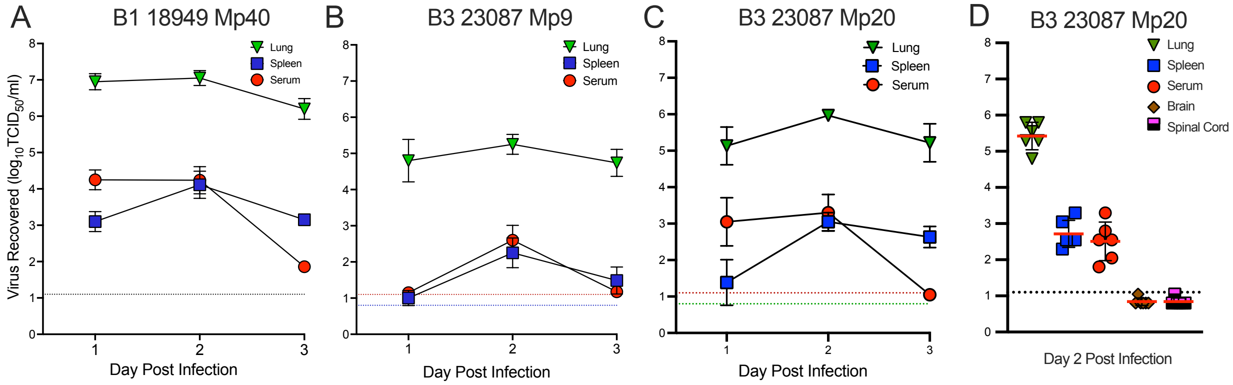

Figure S2. Replication of mouse-adapted EV-D68 strains in AG129 mice. Mice were infected intranasally with the indicated virus then two days post infection, lungs, spleen and serum were assessed for viral load as described in the methods. A, Replication of B1 subclade 18949 Mp40 virus, demonstrating peak titers at 2 days post intranasal infection with $10^{4.5}$ $TCID_{50}$/mouse. B, Replication of B3 subclade 23087 Mp9 virus, demonstrating peak titers at 2 days post intranasal infection with $10^{4.2}$ $TCID_{50}$/mouse. C, Replication of B3 23087 Mp20 virus, demonstrating peak titers 2 days post intranasal infection with $10^{4.4}$ $TCID_{50}$/mouse. D, Replication of B3 23087 Mp20 virus, demonstrating limited CNS dissemination 2 days post intranasal infection with $10^{4.5}$ $TCID_{50}$/mouse. See supplemental table 2 for adaptive mutations and the methods for details on adaptation.

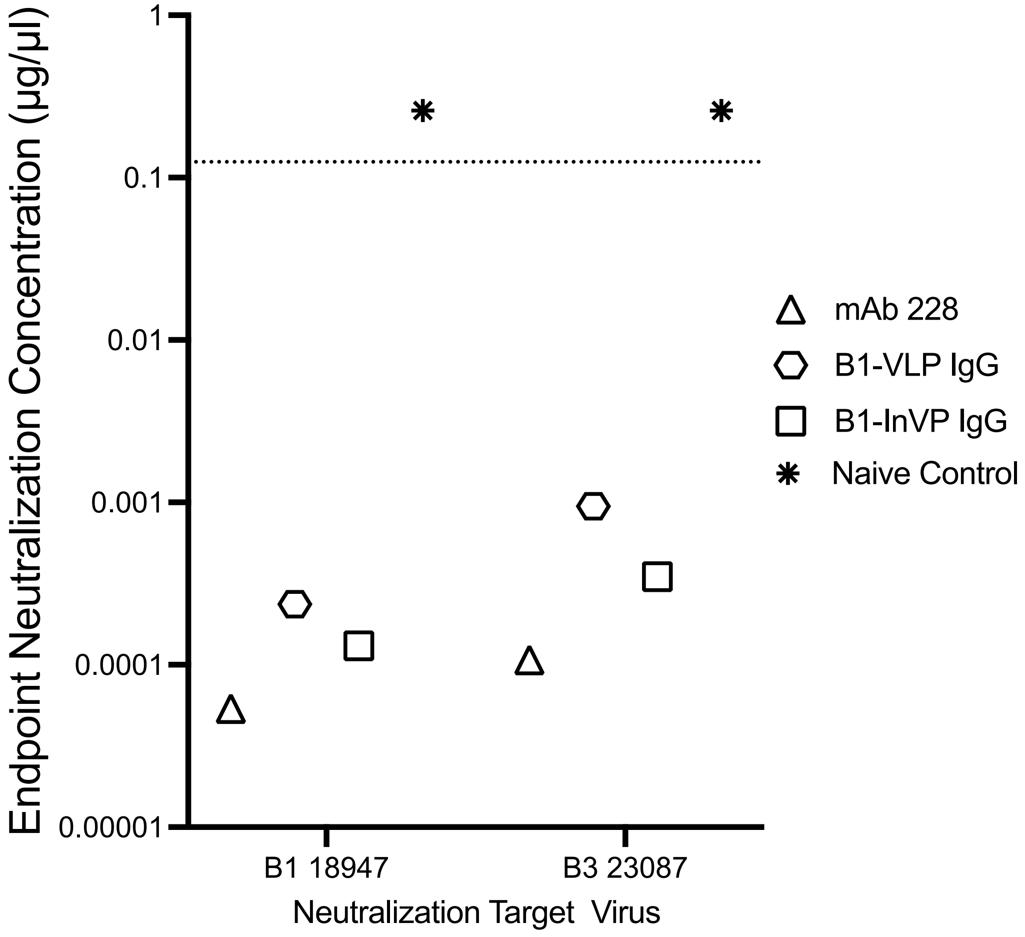

Figure S3. Neutralization capacity of IgG purified from B1 immunogen-vaccinated mouse sera. IgG was purified from pooled sera of mice vaccinated with B1 InVP or VLP as described in figure 2A. The IgG was used in a neutralization assay against US/MO/2014-18947(B1) and USA/2018-23087(B3). mAb 228 and naïve mouse IgG were used as positive and negative controls, respectively. Dashed line indicates the lower limit of detection in the assay.

.

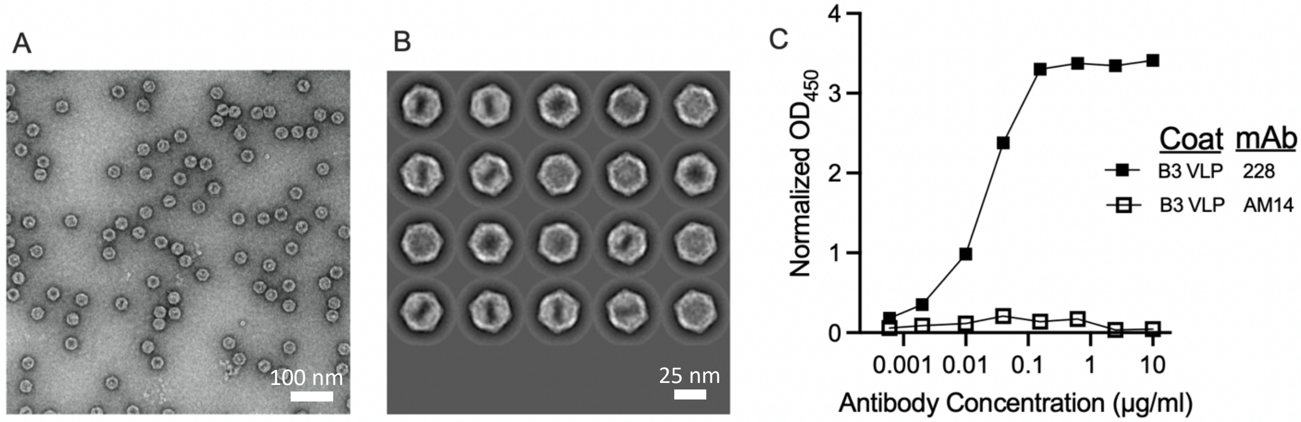

Figure S4. Quality control of B3 VLP. A. Electron micrographs showing the spread (left panel) or 2-D class averages (right panel) of the VLP purified from cells transfected with plasmids expressing the capsid and protease genes from EV-D68 USA/2018-23209. B. ELISA demonstrating binding activity of EVD68-specific mAb 228 or RSV-specific mAb AM14 to B3 VLP adsorbed to assay plates.

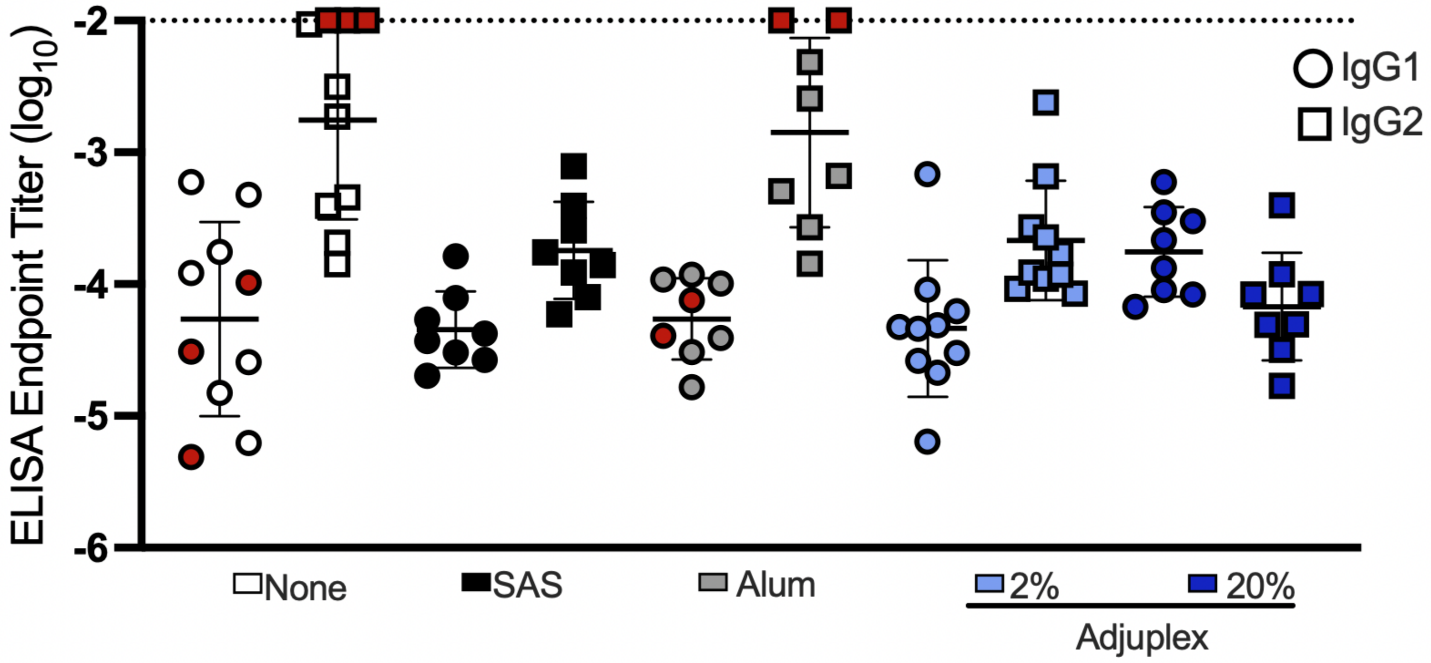

Figure S5. EVD68-specific IgG1 and IgG2a profiles in sera from mice vaccinated with various formulations of B3 VLP. Mice were vaccinated as described in the figure 4A schema and the IgG profiles of the 6 weeks post prime sera was performed as described in the methods. The dotted line indicates the lower limit of detection in the assay. Red symbols indicate serum samples were below the limit of detection for IgG2a secondary antibody and therefore were not included in the IgG1/IgG2a ratio analysis in figure 4B.

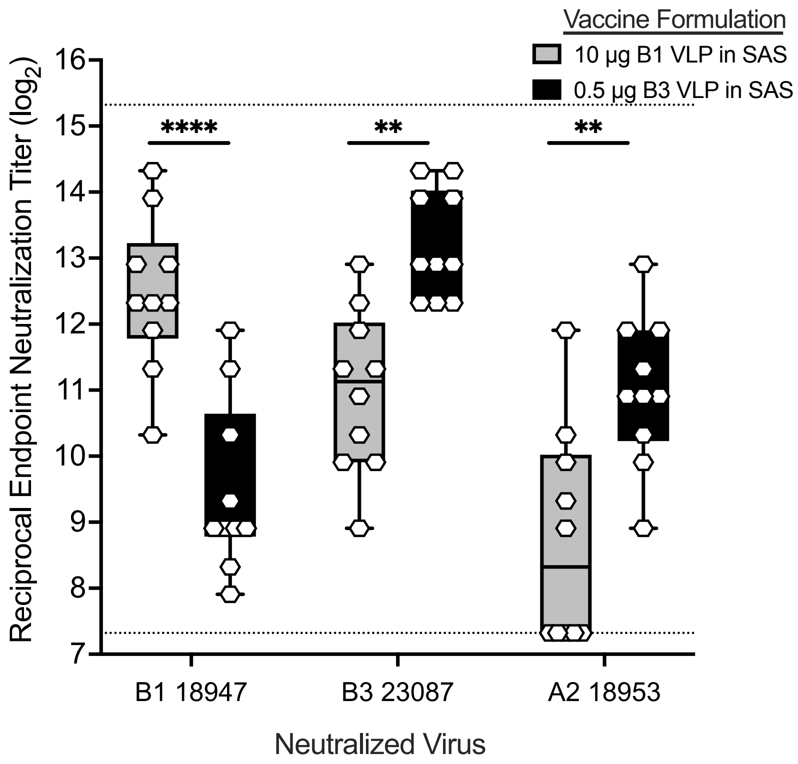

Figure S6. B3 VLP vaccination elicits significantly stronger A2 subclade virus neutralization compared to vaccination with B1 VLP. Cross-neutralization of EV-D68 viruses with sera raised against B1 VLP or B3 VLP formulated with SAS. ANOVA with Tukey's multiple comparisons test was used to compare groups. Dashed lines indicate the upper and lower limit of detection in the neutralization assay. B3:US/2018/23087; B1: US/MO/2014/18947; A2: US/KY/2014/18953.

Table S1. Viruses and VLP used in this study and amino acid sequences of selected variable regions.

Clade	Virus	Accession	Source[†]	VP2 EF*	VP1 BC*	VP1 DE*	VP1 C-Terminus*
B1	US/CO/2014-93	KP126911	VLP	HNTNTSPGFDDIMKGEEGGTF	DHTSSAAQADKNFF	NGSSNNTYV	SAIIGNRDSVKTMPHNI--VNT
B1	US/MO/2014-18947	KM851225	NR-49129	HNTNTSPGFDDIMKGEEGGTF	DHTSSTARADKNFF	NGSGNNTYV	SAIIGNRDSVKTMPHNI--VNT
B2	US/IL/2014-18952	KM851230	NR-49131	HNTNTSPGFDDIMKGEEGGTF	DHTSSAAQTDKNFF	NGSSNNTYV	NAIIGNRDSVKTMPHNI--VTT
B3	USA/2018-23209	MN246002	VLP	HNTTTSPGFDDIMKGEAGGTF	DHTSSTAQTDKNFF	NGSNNNTYV	NAIIGNRDSVKTMPHNI--V
B3	USA/2018-23087	MK491180	NR-51996	HNTTTSPGFDDIMKGEAGGTF	DHTSSTAQTDKNFF	NGSNNNTYV	NAIIGNRDSVKTMPHNI--VTT
B3	USA/2018-23088	MK491181	NR-51997	HNTTTSPGFDDIMKGEAGGTF	DHTSSTARTDKNFF	NGSNNNTYV	NAIIGNRDSVKTMPHNI--VTT
A2	US/KY/2014-18953	KM851231	NR-49132	HNTTTSPGFDDIMKGEEGGTF	NHTSSEARTDKNFY	-GSNNSTYM	NAIIGNRESVKTMPHDIRLVNT

[†]The viruses used in this study were obtained through BEI Resources, NIAID, NIH.

[*]Shaded amino acids in variable loops indicate variation from the B1 US/CO/2014-93 sequence

Table S2. Mouse-adapted EV-D68 used in this study and amino acid sequences of selected variable regions.

Clade	Virus	Accession	Source	VP2 EF	VP1 BC	VP1 DE	VP1 C-Terminus	Other ORF Mutations
B1	US/MO/2014-18949	KM851227	BEI NR-49130	HNTNTSPGFDDIMKGEEGGTF	DHTSSAAQADKNFF	NGSSNNTYA	SAIIGNRDSVKTMPHNIVNT	
	US/MO/2014-18949 Mouse Adapted p30	MH708882	{Evans, 2019 Ref #38}	HNTNTSPGFDDIMKGEEGGTF	DHTSSAAQADKNFF	NGSSNNTYA	SAIIGNRDSVKTMPHNIVNT	
	US/MO/2014-18949 Mouse Adapted p40†		This Manuscript	HNTNTSPGFDDIMKGEEGGTF	DHTSSAAQADKNFF	NGSSNNTYA	SAIIGNRDSVKTMPHNIVNT	None
B3	USA/2018-23087	MK491180	BEI NR-51996	HNTTTSPGFDDIMKGEAGGTF	DHTSSTAQTDKNFF	NGSNNNTYV	NAIIGNRDSVKTMPHNIVTT	
	USA/2018-23087 Mouse Adapted p9††		This Manuscript	HNTTTSPGFDDIMKGEAGGTF	DHTSSTAQTDKNFF	NGSNNNTYV	NAIIGNRDSVKTMPHNIVTT	VP1/P24H
	USA/2018-23087 Mouse Adapted p20††	OQ653554	This Manuscript	HNTTTSPEFDDIMKGEAGGTF	DHTSSTARTDKNFF	NGSNNNTYV	NAIIGNRDSVKTMPHNIVTT	VP1/G207C 2A/T142A VP1/H24P(revert)

†Challenge stock made from 10th additional serial *in vivo* passage in AG129 mouse lungs, total 40 lung passages.
††B3 Subclade Mouse-adapted EV-D68 viruses described in the methods and the legend for Figure S2.
Shaded amino acids indicate mutations after mouse adaptation in variable loops relative to the parental virus.
